# Supplementary material for: Unveiling the anti-obesity potential of Kemuning (Murraya paniculata): A network pharmacology approach
Source: PLoS One. 2024 Aug 29;19(8):e0305544. doi: 10.1371/journal.pone.0305544 (PMC11361609; doi:10.1371/journal.pone.0305544)
Supplement: S1 Table — (PDF) [file pone.0305544.s001.pdf]

S1 Table. Prediction results of active compounds in *M. paniculata* leaves using LC-MS

No	Compound prediction	MS2	Formula	Calc. MW	RT [min]	EtOH p.a	EtOH 50	Water
1	4-aminobenzoic acid	138; 92	C7H7NO2	137.0476	1.087	✓	✓	✓
2	Citric acid	191; 111; 87	C6H8O7	192.0264	1.258			✓
3	L-phenylalanine	91; 103;120	C9H11NO2	165.0787	2.221			✓
4	Trans-3-indoleacrylic acid	189; 146; 118	C11H9NO2	187.0631	4.693	✓	✓	✓
5	DL-tryptophan	188; 170; 146; 118	C11H12N2O2	204.0895	4.699	✓	✓	✓
6	4-O-feruloyl-D-quinic acid	367; 173; 93	C17H20O9	368.1104	8.687	✓	✓	✓
7	Murrangatin	259; 231; 189; 131	C15H16O5	293.1256	12.938	✓	✓	✓
8	Alpha-lapachone	159; 187; 243	C15H14O3	242.0938	14.823	✓	✓	✓
9	Hainanmurpanin	231;203; 189; 131	C17H18O6	335.1356	15.914	✓	✓	✓
10	Murraol	261;243; 189; 131	C15H18O5	260.1041	16.228	✓	✓	✓
11	Murralongin	259; 231; 189; 131	C15H14O4	258.0887	17.746	✓	✓	✓
12	(1R,9S)-5-[(E)-2-(4-chlorophenyl)ethenyl]-11-(pyrimidine-5-carbonyl)-7,11-diazatricyclo[7.3.1.02,7]trideca-2,4-dien-6-one	433	C24H21ClN4O2	432.1411	18.361	✓	✓	✓
13	Paniculatin	259; 231; 189; 131	C20H24O6	360.1565	21.406	✓	✓	✓
14	Limonene, (+/-)-	136; 121; 107; 93	C10H16	136.1252	24.519	✓		
15	Pheophorbide a	593; 533; 461	C35H36N4O5	592.2671	27.489	✓	✓	
16	SB236057	535	C33H34N4O3	534.2617	28.382	✓	✓	✓
