# Supplementary material for: Unveiling the anti-obesity potential of Kemuning (Murraya paniculata): A network pharmacology approach
Source: PLoS One. 2024 Aug 29;19(8):e0305544. doi: 10.1371/journal.pone.0305544 (PMC11361609; doi:10.1371/journal.pone.0305544)
Supplement: S2 Table — (PDF) [file pone.0305544.s002.pdf]

S2 Table. Results of drug-likeness of the compounds using SwissADME

No	Compounds	Molecular Weight	#H-bond acceptors	#H-bond donors	MLOGP	Molar refractivity	GI absorption	Bioavailability Score
1	4-aminobenzoic acid	137.14	2	2	-0.12	37.81*	High	0.85
2	Citric acid	192.12	7	4	-1.48	37.47*	Low*	0.56
3	L-phenylalanine	165.19	3	2	-1.11	45.5	High	0.55
4	trans-3-indoleacrylic acid	187.19	2	2	1.32	54.97	High	0.85
5	DL-tryptophan	204.23	3	3	-1.66	57.36	High	0.55
6	4-O-feruloyl-D-quinic acid	368.34	9	5	-0.81	87.97	Low*	0.11*
7	Murrangatin	276.28	5	2	0.95	75.02	High	0.55
8	alpha-lapachone	242.27	3	0	1.4	67.29	High	0.85
9	Hainanmurpanin	318.32	6	0	1.36	84.27	High	0.55
10	Murraol	260.29	4	1	1.78	74.69	High	0.55
11	Murralongin	258.27	4	0	1.7	73.69	High	0.55
12	(1R,9S)-5-[(E)-2-(4-Chlorophenyl)vinyl]-11-(5-pyrimidinylcarbonyl)-7,11-diazatricyclo[7.3.1.0 2,7]trideca-2,4-dien-6-one	432.9	4	0	2.88	124.43	High	0.55
13	Paniculatin	594.52*	15*	11*	-4.51	139.23*	Low*	0.17*
14	Limonene, (+/-)-	136.23	0	0	3.27	47.12	Low*	0.55
15	Pheophorbide a	592.68*	8	3	1.76	185.79*	Low*	0.56
16	SB236057	534.65*	6	0	4.54	162.77*	High	0.17*

* Value does not meet the minimum requirements.
