# Supplementary material for: Unveiling the anti-obesity potential of Kemuning (Murraya paniculata): A network pharmacology approach
Source: PLoS One. 2024 Aug 29;19(8):e0305544. doi: 10.1371/journal.pone.0305544 (PMC11361609; doi:10.1371/journal.pone.0305544)
Supplement: S3 Table — (PDF) [file pone.0305544.s003.pdf]

S3 Table. Gene Ontology: Biological Processes of the PPARG, EP300, ad PPARGC1A

Term	Overlap	P-value	Adjusted P-value	Old P-value	Old Adjusted P-value	Odds Ratio	Combined Score	Genes
Fat Cell Differentiation (GO:0045444)	3/62	2.836517027605735E-8	7.289848760946739E-6	0	0	59814.0	1039453.9032742921	EP300;PPARG;PPARGC1A
Positive Regulation Of DNA-binding Transcription Factor Activity (GO:0051091)	3/246	1.8383578695824376E-6	2.3622898624134323E-4	0	0	59262.0	782651.7720894068	EP300;PPARG;PPARGC1A
Regulation Of Gluconeogenesis (GO:0006111)	2/31	6.968136336812062E-6	5.969370128535667E-4	0	0	1377.103448275862	16351.950471819193	EP300;PPARGC1A
Negative Regulation Of Smooth Muscle Cell Proliferation (GO:0048662)	2/37	9.978211166975307E-6	6.411000674781635E-4	0	0	1140.6857142857143	13135.117740244841	PPARG;PPARGC1A
Positive Regulation Of Gene Expression (GO:0010628)	3/480	1.3738959782215341E-5	6.902673685811243E-4	0	0	58560.0	655595.3029048318	EP300;PPARG;PPARGC1A
Regulation Of Mitochondrion Organization (GO:0010821)	2/50	1.8345472263811385E-5	6.902673685811243E-4	0	0	831.2083333333334	9065.264276038262	EP300;PPARGC1A
Regulation Of Smooth Muscle Cell Proliferation (GO:0048660)	2/54	2.1427678773293076E-5	6.902673685811243E-4	0	0	767.1153846153846	8247.1248435323	PPARG;PPARGC1A
Positive Regulation Of Nucleic Acid-Templated Transcription (GO:1903508)	3/557	2.1486921979178967E-5	6.902673685811243E-4	0	0	58329.0	626923.9468438141	EP300;PPARG;PPARGC1A
Glucose Homeostasis (GO:0042593)	2/87	5.595622444541673E-5	0.0015978610758302335	0	0	468.5176470588235	4587.2285832395155	PPARG;PPARGC1A
Positive Regulation Of Transmembrane Receptor Protein Serine/Threonine Kinase Signaling Pathway (GO:0090100)	2/95	6.676774316612702E-5	0.0017159309993694644	0	0	428.0430107526882	4115.329843645542	EP300;PPARG
Regulation Of Transforming Growth Factor Beta Receptor Signaling Pathway (GO:0017015)	2/111	9.124302793162746E-5	0.00213176892531166	0	0	364.91743119266056	3394.456097024397	EP300;PPARG
Positive Regulation Of Transcription By RNA Polymerase II (GO:0045944)	3/938	1.0284329378180761E-4	0.0022025605418270464	0	0	57186.0	525099.2449996796	EP300;PPARG;PPARGC1A
Positive Regulation Of DNA-templated Transcription (GO:0045893)	3/1243	2.3951089438663774E-4	0.0047349461428743	0	0	56271.0	469126.3556880545	EP300;PPARG;PPARGC1A
Transcription By RNA Polymerase II (GO:0006366)	2/192	2.733027382147016E-4	0.005017057408655594	0	0	208.49473684210525	1710.6848141465343	PPARG;PPARGC1A
Protein Stabilization (GO:0050821)	2/208	3.2070845874214904E-4	0.00549480492644882	0	0	192.14563106796118	1545.8073892911677	EP300;PPARGC1A
Negative Regulation Of Sequestering Of Triglyceride (GO:0010891)	1/5	7.498314718703743E-4	0.007707135151093626	0	0	2499.125	17982.858997820545	PPARG
Regulation Of DNA-templated Transcription (GO:0006355)	3/1922	8.862309372402333E-4	0.007707135151093626	0	0	54234.0	381185.45816936734	EP300;PPARG;PPARGC1A

Regulation Of Cellular Response To Insulin Stimulus (GO:1900076)	1/6	8.997538788767587E-4	0.007707135151093626	0	0	1999.2	14021.167888467728	PPARG
Macrophage Derived Foam Cell Differentiation (GO:0010742)	1/6	8.997538788767587E-4	0.007707135151093626	0	0	1999.2	14021.167888467728	PPARG
Regulation Of Vascular Associated Smooth Muscle Cell Apoptotic Process (GO:1905459)	1/6	8.997538788767587E-4	0.007707135151093626	0	0	1999.2	14021.167888467728	PPARG
White Fat Cell Differentiation (GO:0050872)	1/6	8.997538788767587E-4	0.007707135151093626	0	0	1999.2	14021.167888467728	PPARG
Negative Regulation Of Response To Type II Interferon (GO:0060331)	1/6	8.997538788767587E-4	0.007707135151093626	0	0	1999.2	14021.167888467728	PPARG
Negative Regulation Of Type II Interferon-Mediated Signaling Pathway (GO:0060336)	1/6	8.997538788767587E-4	0.007707135151093626	0	0	1999.2	14021.167888467728	PPARG
Regulation Of Adiponectin Secretion (GO:0070163)	1/6	8.997538788767587E-4	0.007707135151093626	0	0	1999.2	14021.167888467728	PPARG
Regulation Of Cardiac Muscle Hypertrophy In Response To Stress (GO:1903242)	1/6	8.997538788767587E-4	0.007707135151093626	0	0	1999.2	14021.167888467728	PPARG
Positive Regulation Of Macromolecule Metabolic Process (GO:0010604)	2/364	9.790570835035576E-4	0.007707135151093626	0	0	108.48066298342542	751.6539014414852	PPARG;PPARGC1A
Regulation Of Transcription By RNA Polymerase II (GO:0006357)	3/2028	0.0010411817181225926	0.007707135151093626	0	0	53916.0	370262.6814359409	EP300;PPARG;PPARGC1A
Positive Regulation Of Gluconeogenesis (GO:0045722)	1/7	0.001049661432299498	0.007707135151093626	0	0	1665.9166666666667	11427.001554688255	PPARGC1A
Peroxisome Proliferator Activated Receptor Signaling Pathway (GO:0035357)	1/7	0.001049661432299498	0.007707135151093626	0	0	1665.9166666666667	11427.001554688255	PPARG
Foam Cell Differentiation (GO:0090077)	1/7	0.001049661432299498	0.007707135151093626	0	0	1665.9166666666667	11427.001554688255	PPARG
Negative Regulation Of miRNA-mediated Gene Silencing (GO:0060965)	1/7	0.001049661432299498	0.007707135151093626	0	0	1665.9166666666667	11427.001554688255	PPARG
Positive Regulation Of Adipose Tissue Development (GO:1904179)	1/7	0.001049661432299498	0.007707135151093626	0	0	1665.9166666666667	11427.001554688255	PPARG
Negative Regulation Of SMAD Protein Signal Transduction (GO:0060392)	1/7	0.001049661432299498	0.007707135151093626	0	0	1665.9166666666667	11427.001554688255	PPARG
Negative Regulation Of Cell Population Proliferation (GO:0008285)	2/379	0.0010609901783486244	0.007707135151093626	0	0	104.08488063660478	712.830787853366	PPARG;PPARGC1A
Positive Regulation Of Multicellular Organismal Process (GO:0051240)	2/387	0.00110601604120992	0.007707135151093626	0	0	101.88051948051948	693.4997661569278	PPARG;PPARGC1A
Positive Regulation Of ATP Biosynthetic Process (GO:2001171)	1/8	0.0011995541091196305	0.007707135151093626	0	0	1427.857142857143	9603.48923469958	PPARGC1A

Positive Regulation Of Smooth Muscle Cell Apoptotic Process (GO:0034393)	1/8	0.0011995541091196305	0.007707135151093626	0	0	1427.857142857143	9603.48923469958	PPARG
Internal Protein Amino Acid Acetylation (GO:0006475)	1/8	0.0011995541091196305	0.007707135151093626	0	0	1427.857142857143	9603.48923469958	EP300
Negative Regulation Of Cholesterol Storage (GO:0010887)	1/8	0.0011995541091196305	0.007707135151093626	0	0	1427.857142857143	9603.48923469958	PPARG
Brown Fat Cell Differentiation (GO:0050873)	1/8	0.0011995541091196305	0.007707135151093626	0	0	1427.857142857143	9603.48923469958	PPARGC1A
Peptidyl-Lysine Acetylation (GO:0018394)	1/9	0.0013494318923464072	0.007881909007568787	0	0	1249.3125	8255.5464452602	EP300
Negative Regulation Of Lipid Localization (GO:1905953)	1/9	0.0013494318923464072	0.007881909007568787	0	0	1249.3125	8255.5464452602	PPARG
Positive Regulation Of ATP Metabolic Process (GO:1903580)	1/9	0.0013494318923464072	0.007881909007568787	0	0	1249.3125	8255.5464452602	PPARGC1A
Regulation Of RNA Polymerase II Regulatory Region Sequence-Specific DNA Binding (GO:1903025)	1/9	0.0013494318923464072	0.007881909007568787	0	0	1249.3125	8255.5464452602	EP300
Negative Regulation Of Pathway-Restricted SMAD Protein Phosphorylation (GO:0060394)	1/10	0.0014992947695318503	0.008138521386479771	0	0	1110.4444444444443	7220.954198683598	PPARG
Monocyte Differentiation (GO:0030224)	1/10	0.0014992947695318503	0.008138521386479771	0	0	1110.4444444444443	7220.954198683598	PPARG
Regulation Of Type II Interferon-Mediated Signaling Pathway (GO:0060334)	1/11	0.0016491427308511233	0.008138521386479771	0	0	999.35	6403.334808352065	PPARG
Regulation Of Adipose Tissue Development (GO:1904177)	1/11	0.0016491427308511233	0.008138521386479771	0	0	999.35	6403.334808352065	PPARG
Retinoic Acid Receptor Signaling Pathway (GO:0048384)	1/11	0.0016491427308511233	0.008138521386479771	0	0	999.35	6403.334808352065	PPARG
Lipoprotein Transport (GO:0042953)	1/11	0.0016491427308511233	0.008138521386479771	0	0	999.35	6403.334808352065	PPARG
Negative Regulation Of Macrophage Derived Foam Cell Differentiation (GO:0010745)	1/12	0.0017989757687595922	0.008138521386479771	0	0	908.4545454545455	5741.921288120221	PPARG
Positive Regulation Of Purine Nucleotide Biosynthetic Process (GO:1900373)	1/12	0.0017989757687595922	0.008138521386479771	0	0	908.4545454545455	5741.921288120221	PPARGC1A
Negative Regulation Of Cardiac Muscle Hypertrophy (GO:0010614)	1/12	0.0017989757687595922	0.008138521386479771	0	0	908.4545454545455	5741.921288120221	PPARG
Lipoprotein Localization (GO:0044872)	1/12	0.0017989757687595922	0.008138521386479771	0	0	908.4545454545455	5741.921288120221	PPARG
Positive Regulation Of Fatty Acid Oxidation (GO:0046321)	1/12	0.0017989757687595922	0.008138521386479771	0	0	908.4545454545455	5741.921288120221	PPARGC1A
Lipid Oxidation (GO:0034440)	1/13	0.001948793877001376	0.008138521386479771	0	0	832.7083333333334	5196.553511599695	PPARGC1A

Positive Regulation Of Intracellular Signal Transduction (GO:1902533)	2/525	0.002027340473602929	0.008138521386479771	0	0	74.47036328871893	461.79299085255496	EP300;PPARG
Regulation Of Cholesterol Storage (GO:0010885)	1/14	0.0020985970505495682	0.008138521386479771	0	0	768.6153846153846	4739.656184381286	PPARG
Positive Regulation Of Histone Acetylation (GO:0035066)	1/14	0.0020985970505495682	0.008138521386479771	0	0	768.6153846153846	4739.656184381286	PPARGC1A
Regulation Of Sequestering Of Triglyceride (GO:0010889)	1/14	0.0020985970505495682	0.008138521386479771	0	0	768.6153846153846	4739.656184381286	PPARG
Regulation Of ATP Biosynthetic Process (GO:2001169)	1/14	0.0020985970505495682	0.008138521386479771	0	0	768.6153846153846	4739.656184381286	PPARGC1A
Negative Regulation Of Receptor Signaling Pathway Via STAT (GO:1904893)	1/14	0.0020985970505495682	0.008138521386479771	0	0	768.6153846153846	4739.656184381286	PPARG
Respiratory Electron Transport Chain (GO:0022904)	1/14	0.0020985970505495682	0.008138521386479771	0	0	768.6153846153846	4739.656184381286	PPARGC1A
Positive Regulation Of Transporter Activity (GO:0032411)	1/14	0.0020985970505495682	0.008138521386479771	0	0	768.6153846153846	4739.656184381286	PPARGC1A
Electron Transport Chain (GO:0022900)	1/14	0.0020985970505495682	0.008138521386479771	0	0	768.6153846153846	4739.656184381286	PPARGC1A
Regulation Of Carbohydrate Catabolic Process (GO:0043470)	1/14	0.0020985970505495682	0.008138521386479771	0	0	768.6153846153846	4739.656184381286	EP300
Regulation Of Cellular Response To Heat (GO:1900034)	1/15	0.0022483852857590027	0.008138521386479771	0	0	713.6785714285714	4351.685756891909	EP300
Positive Regulation Of Glucose Metabolic Process (GO:0010907)	1/15	0.0022483852857590027	0.008138521386479771	0	0	713.6785714285714	4351.685756891909	PPARGC1A
Negative Regulation Of Gluconeogenesis (GO:0045721)	1/15	0.0022483852857590027	0.008138521386479771	0	0	713.6785714285714	4351.685756891909	EP300
N-terminal Protein Amino Acid Acetylation (GO:0006474)	1/15	0.0022483852857590027	0.008138521386479771	0	0	713.6785714285714	4351.685756891909	EP300
Regulation Of miRNA-mediated Gene Silencing (GO:0060964)	1/15	0.0022483852857590027	0.008138521386479771	0	0	713.6785714285714	4351.685756891909	PPARG
Regulation Of Fatty Acid Oxidation (GO:0046320)	1/16	0.0023981585785517907	0.008288793038627243	0	0	666.0666666666667	4018.4162309154813	PPARGC1A
Positive Regulation By Host Of Viral Transcription (GO:0043923)	1/16	0.0023981585785517907	0.008288793038627243	0	0	666.0666666666667	4018.4162309154813	EP300
Regulation Of Receptor Signaling Pathway Via STAT (GO:1904892)	1/17	0.0025479169262706313	0.008288793038627243	0	0	624.40625	3729.2533063250903	PPARG
Regulation Of Tubulin Deacetylation (GO:0090043)	1/17	0.0025479169262706313	0.008288793038627243	0	0	624.40625	3729.2533063250903	EP300
Positive Regulation Of Peptidyl-Lysine Acetylation (GO:2000758)	1/17	0.0025479169262706313	0.008288793038627243	0	0	624.40625	3729.2533063250903	PPARGC1A
Positive Regulation Of SMAD Protein Signal Transduction (GO:0060391)	1/17	0.0025479169262706313	0.008288793038627243	0	0	624.40625	3729.2533063250903	PPARG
Negative Regulation Of Vascular Associated Smooth Muscle Cell Proliferation (GO:1904706)	1/17	0.0025479169262706313	0.008288793038627243	0	0	624.40625	3729.2533063250903	PPARG

Positive Regulation Of Fatty Acid Metabolic Process (GO:0045923)	1/17	0.0025479169262706313	0.008288793038627243	0	0	624.40625	3729.2533063250903	PPARGC1A
Regulation Of Protein Deacetylation (GO:0090311)	1/18	0.0026976603266888493	0.008454862243402857	0	0	587.6470588235294	3476.150033460634	EP300
Negative Regulation Of Lipid Storage (GO:0010888)	1/18	0.0026976603266888493	0.008454862243402857	0	0	587.6470588235294	3476.150033460634	PPARG
Regulation Of Vascular Endothelial Cell Proliferation (GO:1905562)	1/18	0.0026976603266888493	0.008454862243402857	0	0	587.6470588235294	3476.150033460634	PPARG
Positive Regulation Of Hormone Secretion (GO:0046887)	1/19	0.002847388777574066	0.008509057160889942	0	0	554.9722222222222	3252.888056951129	PPARG
Stimulatory C-type Lectin Receptor Signaling Pathway (GO:0002223)	1/19	0.002847388777574066	0.008509057160889942	0	0	554.9722222222222	3252.888056951129	EP300
Cellular Response To Lectin (GO:1990858)	1/19	0.002847388777574066	0.008509057160889942	0	0	554.9722222222222	3252.888056951129	EP300
Intrinsic Apoptotic Signaling Pathway In Response To DNA Damage By P53 Class Mediator (GO:0042771)	1/19	0.002847388777574066	0.008509057160889942	0	0	554.9722222222222	3252.888056951129	EP300
Negative Regulation Of miRNA Transcription (GO:1902894)	1/20	0.0029971022769704784	0.008853509025073713	0	0	525.7368421052631	3054.5885497256336	PPARG
Negative Regulation Of miRNA Metabolic Process (GO:2000629)	1/21	0.0031468008242819096	0.008917857845377521	0	0	499.425	2877.3716896348615	PPARG
Regulation Of Lipid Storage (GO:0010883)	1/21	0.0031468008242819096	0.008917857845377521	0	0	499.425	2877.3716896348615	PPARG
Positive Regulation Of Transcription Regulatory Region DNA Binding (GO:2000679)	1/21	0.0031468008242819096	0.008917857845377521	0	0	499.425	2877.3716896348615	EP300
Placenta Development (GO:0001890)	1/22	0.0032964844175520022	0.008917857845377521	0	0	475.6190476190476	2718.114679972292	PPARG
Cellular Response To Low-Density Lipoprotein Particle Stimulus (GO:0071404)	1/22	0.0032964844175520022	0.008917857845377521	0	0	475.6190476190476	2718.114679972292	PPARG
Regulation Of Histone Acetylation (GO:0035065)	1/22	0.0032964844175520022	0.008917857845377521	0	0	475.6190476190476	2718.114679972292	PPARGC1A
Regulation Of Intracellular Steroid Hormone Receptor Signaling Pathway (GO:0033143)	1/22	0.0032964844175520022	0.008917857845377521	0	0	475.6190476190476	2718.114679972292	EP300
Positive Regulation Of Cholesterol Efflux (GO:0010875)	1/22	0.0032964844175520022	0.008917857845377521	0	0	475.6190476190476	2718.114679972292	PPARG
Regulation Of Cellular Response To Transforming Growth Factor Beta Stimulus (GO:1903844)	1/23	0.0034461530563075866	0.009130529231660307	0	0	453.97727272727275	2574.27663877793	PPARG
Long-Chain Fatty Acid Transport (GO:0015909)	1/23	0.0034461530563075866	0.009130529231660307	0	0	453.97727272727275	2574.27663877793	PPARG
Mononuclear Cell Differentiation (GO:1903131)	1/24	0.0035958067387344135	0.009241223318547442	0	0	434.2173913043478	2443.7697931466887	PPARG

Positive Regulation Of Cellular Response To Transforming Growth Factor Beta Stimulus (GO:1903846)	1/24	0.0035958067387344135	0.009241223318547442	0	0	434.2173913043478	2443.7697931466887	EP300
Positive Regulation Of Transforming Growth Factor Beta Receptor Signaling Pathway (GO:0030511)	1/24	0.0035958067387344135	0.009241223318547442	0	0	434.2173913043478	2443.7697931466887	EP300
Negative Regulation Of Signaling Receptor Activity (GO:2000272)	1/25	0.0037454454654532016	0.009530489946747255	0	0	416.1041666666667	2324.8633249285726	PPARG
Regulation Of SMAD Protein Signal Transduction (GO:0060390)	1/27	0.004044678046227062	0.009995021710388028	0	0	384.0576923076923	2116.2935816693675	PPARG
Regulation Of Mitochondrial Fission (GO:0090140)	1/27	0.004044678046227062	0.009995021710388028	0	0	384.0576923076923	2116.2935816693675	PPARG
Regulation Of Androgen Receptor Signaling Pathway (GO:0060765)	1/27	0.004044678046227062	0.009995021710388028	0	0	384.0576923076923	2116.2935816693675	EP300
Positive Regulation Of Protein Import Into Nucleus (GO:0042307)	1/28	0.004194271900919973	0.010265979795585077	0	0	369.81481481481484	2024.379431258311	EP300
Negative Regulation Of Transcription By RNA Polymerase II (GO:0000122)	2/763	0.00425007148382476	0.010304418597575126	0	0	50.55453350854139	276.069181206984	EP300;PPARG
Protein Acetylation (GO:0006473)	1/29	0.00434385079686094	0.010433361259750107	0	0	356.5892857142857	1939.4870011141588	EP300
Regulation Of Macrophage Derived Foam Cell Differentiation (GO:0010743)	1/30	0.004493414734230119	0.010498250788155822	0	0	344.2758620689655	1860.860040913568	PPARG
Innate Immune Response Activating Cell Surface Receptor Signaling Pathway (GO:0002220)	1/30	0.004493414734230119	0.010498250788155822	0	0	344.2758620689655	1860.860040913568	EP300
Negative Regulation Of Carbohydrate Metabolic Process (GO:0045912)	1/30	0.004493414734230119	0.010498250788155822	0	0	344.2758620689655	1860.860040913568	EP300
Positive Regulation Of Cholesterol Transport (GO:0032376)	1/31	0.0046429637132384795	0.010749924993714317	0	0	332.78333333333336	1787.845973802925	PPARG
Regulation Of Cholesterol Efflux (GO:0010874)	1/32	0.004792497734793632	0.010804139630192663	0	0	322.03225806451616	1719.8788256956173	PPARG
Gluconeogenesis (GO:0006094)	1/32	0.004792497734793632	0.010804139630192663	0	0	322.03225806451616	1719.8788256956173	PPARGC1A
Negative Regulation Of Osteoblast Differentiation (GO:0045668)	1/32	0.004792497734793632	0.010804139630192663	0	0	322.03225806451616	1719.8788256956173	PPARG
Positive Regulation Of DNA Binding (GO:0043388)	1/33	0.004942016796379297	0.010855541168115209	0	0	311.953125	1656.4654076932638	PPARG
Negative Regulation Of Small Molecule Metabolic Process (GO:0062014)	1/33	0.004942016796379297	0.010855541168115209	0	0	311.953125	1656.4654076932638	EP300
Negative Regulation Of Blood Vessel Endothelial Cell Migration (GO:0043537)	1/33	0.004942016796379297	0.010855541168115209	0	0	311.953125	1656.4654076932638	PPARG

Transcription Initiation-Coupled Chromatin Remodeling (GO:0045815)	1/34	0.005091520900981562	0.010904340596268845	0	0	302.4848484848485	1597.1740514070286	EP300
Positive Regulation Of Organelle Organization (GO:0010638)	1/34	0.005091520900981562	0.010904340596268845	0	0	302.4848484848485	1597.1740514070286	PPARGC1A
Response To Estrogen (GO:0043627)	1/34	0.005091520900981562	0.010904340596268845	0	0	302.4848484848485	1597.1740514070286	EP300
Hexose Biosynthetic Process (GO:0019319)	1/35	0.005241010046096412	0.01103694162506868	0	0	293.5735294117647	1541.6253665904292	PPARGC1A
Intrinsic Apoptotic Signaling Pathway By P53 Class Mediator (GO:0072332)	1/35	0.005241010046096412	0.01103694162506868	0	0	293.5735294117647	1541.6253665904292	EP300
Protein Destabilization (GO:0031648)	1/36	0.005390484233154448	0.01103694162506868	0	0	285.1714285714286	1489.484608802673	EP300
Negative Regulation Of Endothelial Cell Migration (GO:0010596)	1/37	0.005539943461610349	0.01103694162506868	0	0	277.2361111111111	1440.4553417617176	PPARG
Negative Regulation Of Endothelial Cell Proliferation (GO:0001937)	1/37	0.005539943461610349	0.01103694162506868	0	0	277.2361111111111	1440.4553417617176	PPARG
Protein Acylation (GO:0043543)	1/37	0.005539943461610349	0.01103694162506868	0	0	277.2361111111111	1440.4553417617176	EP300
Regulation Of Vascular Associated Smooth Muscle Cell Proliferation (GO:1904705)	1/37	0.005539943461610349	0.01103694162506868	0	0	277.2361111111111	1440.4553417617176	PPARG
Positive Regulation Of Receptor Signaling Pathway Via JAK-STAT (GO:0046427)	1/37	0.005539943461610349	0.01103694162506868	0	0	277.2361111111111	1440.4553417617176	EP300
Positive Regulation Of Receptor Signaling Pathway Via STAT (GO:1904894)	1/37	0.005539943461610349	0.01103694162506868	0	0	277.2361111111111	1440.4553417617176	EP300
Regulation Of Protein Import Into Nucleus (GO:0042306)	1/38	0.005689387732894645	0.011077065510256999	0	0	269.72972972972974	1394.274144706199	EP300
mRNA Transcription By RNA Polymerase II (GO:0042789)	1/38	0.005689387732894645	0.011077065510256999	0	0	269.72972972972974	1394.274144706199	PPARG
Fatty Acid Transport (GO:0015908)	1/38	0.005689387732894645	0.011077065510256999	0	0	269.72972972972974	1394.274144706199	PPARG
Negative Regulation Of Mitochondrion Organization (GO:0010823)	1/39	0.005838817046122731	0.01119832821532494	0	0	262.61842105263156	1350.7061704673467	PPARG
Internal Peptidyl-Lysine Acetylation (GO:0018393)	1/39	0.005838817046122731	0.01119832821532494	0	0	262.61842105263156	1350.7061704673467	EP300
Regulation Of Small Molecule Metabolic Process (GO:0062012)	1/40	0.005988231401979197	0.011315996105210687	0	0	255.87179487179486	1309.541398623503	EP300
Regulation Of Glycolytic Process (GO:0006110)	1/40	0.005988231401979197	0.011315996105210687	0	0	255.87179487179486	1309.541398623503	EP300
Modulation By Host Of Symbiont Process (GO:0051851)	1/42	0.00628701524151983	0.011567488635805305	0	0	243.3658536585366	1233.6869399593022	EP300
Regulation Of Primary Metabolic Process (GO:0080090)	1/43	0.006436384727315792	0.011567488635805305	0	0	237.5595238095238	1198.6750596729298	PPARG
mRNA Transcription (GO:0009299)	1/43	0.006436384727315792	0.011567488635805305	0	0	237.5595238095238	1198.6750596729298	PPARG

Positive Regulation Of miRNA Transcription (GO:1902895)	1/43	0.006436384727315792	0.011567488635805305	0	0	237.5595238095238	1198.6750596729298	PPARG
Negative Regulation Of BMP Signaling Pathway (GO:0030514)	1/43	0.006436384727315792	0.011567488635805305	0	0	237.5595238095238	1198.6750596729298	PPARG
Intracellular Glucose Homeostasis (GO:0001678)	1/43	0.006436384727315792	0.011567488635805305	0	0	237.5595238095238	1198.6750596729298	PPARGC1A
Energy Derivation By Oxidation Of Organic Compounds (GO:0015980)	1/43	0.006436384727315792	0.011567488635805305	0	0	237.5595238095238	1198.6750596729298	PPARGC1A
Positive Regulation Of Nucleocytoplasmic Transport (GO:0046824)	1/44	0.006585739256759462	0.011753715201299874	0	0	232.02325581395348	1165.417705666189	EP300
Regulation Of Cellular Metabolic Process (GO:0031323)	1/45	0.006735078830116054	0.01193734661613673	0	0	226.73863636363637	1133.7897191126328	EP300
Regulation Of Signal Transduction By P53 Class Mediator (GO:1901796)	1/47	0.007033713110919517	0.012381262119906273	0	0	216.8586956521739	1074.9773440238823	EP300
Regulation Of DNA Binding (GO:0051101)	1/48	0.007183007818324503	0.01255804768237685	0	0	212.2340425531915	1047.5951010155554	PPARG
Positive Regulation Of miRNA Metabolic Process (GO:2000630)	1/49	0.007332287573040788	0.01256265270847655	0	0	207.80208333333334	1021.4444344646477	PPARG
Positive Regulation Of Pathway-Restricted SMAD Protein Phosphorylation (GO:0010862)	1/49	0.007332287573040788	0.01256265270847655	0	0	207.80208333333334	1021.4444344646477	PPARG
Intrinsic Apoptotic Signaling Pathway In Response To DNA Damage (GO:0008630)	1/49	0.007332287573040788	0.01256265270847655	0	0	207.80208333333334	1021.4444344646477	EP300
Positive Regulation Of Fat Cell Differentiation (GO:0045600)	1/50	0.007481552373416279	0.012733503046145588	0	0	203.55102040816325	996.4463577649728	PPARG
Negative Regulation Of DNA-templated Transcription (GO:0045892)	2/1025	0.007603827650914257	0.012817752750131726	0	0	37.09481915933529	180.98946274130418	EP300;PPARG
Negative Regulation Of MAP Kinase Activity (GO:0043407)	1/51	0.0076308022208955405	0.012817752750131726	0	0	199.47	972.5284117534744	PPARG
Fatty Acid Oxidation (GO:0019395)	1/52	0.007780037114753103	0.012983568431763294	0	0	195.54901960784315	949.6240090215041	PPARGC1A
Regulation Of Receptor Signaling Pathway Via JAK-STAT (GO:0046425)	1/53	0.007929257057990018	0.013062942717329707	0	0	191.77884615384616	927.6718550806611	EP300
Positive Regulation Of NIK/NF-kappaB Signaling (GO:1901224)	1/53	0.007929257057990018	0.013062942717329707	0	0	191.77884615384616	927.6718550806611	EP300
Regulation Of Lipid Metabolic Process (GO:0019216)	1/54	0.008078462047277996	0.013140283203483828	0	0	188.1509433962264	906.615436737158	PPARG
Regulation Of Blood Vessel Endothelial Cell Migration (GO:0043535)	1/54	0.008078462047277996	0.013140283203483828	0	0	188.1509433962264	906.615436737158	PPARG

Positive Regulation Of Mitochondrion Organization (GO:0010822)	1/58	0.008675132502749212	0.013828265394958452	0	0	174.91228070175438	830.360139497581	PPARGC1A
DNA-templated Transcription Initiation (GO:0006352)	1/59	0.008824262742308117	0.013828265394958452	0	0	171.88793103448276	813.0729238663458	PPARGC1A
Glucose Metabolic Process (GO:0006006)	1/59	0.008824262742308117	0.013828265394958452	0	0	171.88793103448276	813.0729238663458	PPARGC1A
Negative Regulation Of Protein-Containing Complex Assembly (GO:0031333)	1/59	0.008824262742308117	0.013828265394958452	0	0	171.88793103448276	813.0729238663458	EP300
Regulation Of miRNA Transcription (GO:1902893)	1/59	0.008824262742308117	0.013828265394958452	0	0	171.88793103448276	813.0729238663458	PPARG
Negative Regulation Of Cytokine-Mediated Signaling Pathway (GO:0001960)	1/59	0.008824262742308117	0.013828265394958452	0	0	171.88793103448276	813.0729238663458	PPARG
Histone Acetylation (GO:0016573)	1/60	0.00897337803301339	0.013976716087784492	0	0	168.96610169491527	796.4205513462199	EP300
BMP Signaling Pathway (GO:0030509)	1/61	0.009122478376120959	0.01401404032655147	0	0	166.14166666666668	780.3696944503212	PPARG
Regulation Of Pathway-Restricted SMAD Protein Phosphorylation (GO:0060393)	1/61	0.009122478376120959	0.01401404032655147	0	0	166.14166666666668	780.3696944503212	PPARG
Regulation Of Gene Expression (GO:0010468)	2/1127	0.009160929085060884	0.01401404032655147	0	0	33.550222222222224	157.44474040747147	PPARG;PPARGC1A
Regulation Of Cellular Catabolic Process (GO:0031329)	1/63	0.009420634221166173	0.014326053223903588	0	0	160.76612903225808	749.9503377031319	EP300
Cellular Response To Light Stimulus (GO:0071482)	1/65	0.009718730281484593	0.014692433425538472	0	0	155.7265625	721.5902191436588	EP300
Cellular Response To BMP Stimulus (GO:0071773)	1/66	0.009867755893827221	0.014830486928149684	0	0	153.3230769230769	708.1199963366474	PPARG
Activation Of Cysteine-Type Endopeptidase Activity Involved In Apoptotic Process (GO:0006919)	1/67	0.01001676656007227	0.014966912825224263	0	0	150.99242424242425	695.0928600722876	PPARG
Myeloid Leukocyte Differentiation (GO:0002573)	1/71	0.010612659796938678	0.015675020504673794	0	0	142.3357142857143	647.0165481479889	PPARG
Negative Regulation Of Cellular Response To Growth Factor Stimulus (GO:0090288)	1/71	0.010612659796938678	0.015675020504673794	0	0	142.3357142857143	647.0165481479889	PPARG
Cellular Response To Hypoxia (GO:0071456)	1/72	0.010761595752488154	0.01571437561584918	0	0	140.32394366197184	635.9160389879967	PPARG
Positive Regulation Of Protein Localization To Nucleus (GO:1900182)	1/72	0.010761595752488154	0.01571437561584918	0	0	140.32394366197184	635.9160389879967	EP300
Cellular Response To Decreased Oxygen Levels (GO:0036294)	1/73	0.010910516765723988	0.015841823778480594	0	0	138.36805555555554	625.1507650815512	PPARG
Negative Regulation Of Neuron Apoptotic Process (GO:0043524)	1/74	0.011059422838055624	0.015967818367305032	0	0	136.46575342465752	614.7062292455009	PPARGC1A

Regulation Of Fat Cell Differentiation (GO:0045598)	1/75	0.011208313971683172	0.01600298161512542	0	0	134.61486486486487	604.5687465206931	PPARG
Regulation Of BMP Signaling Pathway (GO:0030510)	1/75	0.011208313971683172	0.01600298161512542	0	0	134.61486486486487	604.5687465206931	PPARG
Transcription Initiation At RNA Polymerase II Promoter (GO:0006367)	1/76	0.011357190165993224	0.01603735094868274	0	0	132.81333333333333	594.7253885716618	PPARGC1A
Positive Regulation Of Protein Secretion (GO:0050714)	1/76	0.011357190165993224	0.01603735094868274	0	0	132.81333333333333	594.7253885716618	PPARG
Negative Regulation Of Transforming Growth Factor Beta Receptor Signaling Pathway (GO:0030512)	1/77	0.011506051423331512	0.01607095225976195	0	0	131.05921052631578	585.1639324621284	PPARG
Negative Regulation Of Blood Vessel Morphogenesis (GO:2000181)	1/77	0.011506051423331512	0.01607095225976195	0	0	131.05921052631578	585.1639324621284	PPARG
Cellular Response To UV (GO:0034644)	1/79	0.011803729124474183	0.016222237352886977	0	0	127.68589743589743	566.841082547516	EP300
Response To Insulin (GO:0032868)	1/79	0.011803729124474183	0.016222237352886977	0	0	127.68589743589743	566.841082547516	PPARG
Negative Regulation Of Cellular Component Organization (GO:0051129)	1/79	0.011803729124474183	0.016222237352886977	0	0	127.68589743589743	566.841082547516	EP300
Negative Regulation Of Protein Serine/Threonine Kinase Activity (GO:0071901)	1/80	0.011952545569943683	0.016339384103593226	0	0	126.0632911392405	558.058364527147	PPARG
Histone Modification (GO:0016570)	1/81	0.012101347079866988	0.01645527089696199	0	0	124.48125	549.514822983903	EP300
Epigenetic Regulation Of Gene Expression (GO:0040029)	1/83	0.01239890529576631	0.016683343774931633	0	0	121.4329268292683	533.1084106979374	EP300
Regulation Of NIK/NF-kappaB Signaling (GO:1901222)	1/83	0.01239890529576631	0.016683343774931633	0	0	121.4329268292683	533.1084106979374	EP300
mRNA Metabolic Process (GO:0016071)	1/85	0.012696403776118125	0.016819462734342053	0	0	118.5297619047619	517.552677869987	PPARGC1A
Cellular Respiration (GO:0045333)	1/85	0.012696403776118125	0.016819462734342053	0	0	118.5297619047619	517.552677869987	PPARGC1A
Regulation Of Osteoblast Differentiation (GO:0045667)	1/85	0.012696403776118125	0.016819462734342053	0	0	118.5297619047619	517.552677869987	PPARG
Negative Regulation Of Angiogenesis (GO:0016525)	1/86	0.012845130618796025	0.01692922343092604	0	0	117.12941176470588	510.0740472374152	PPARG
Negative Regulation Of Neuron Death (GO:1901215)	1/88	0.013142539507388676	0.017232819660198417	0	0	114.42528735632185	495.67901892070734	PPARGC1A
Cellular Response To Chemical Stress (GO:0062197)	1/89	0.013291221555431713	0.01725173706942399	0	0	113.11931818181819	488.7491512268038	PPARGC1A
Cellular Response To Peptide Hormone Stimulus (GO:0071375)	1/89	0.013291221555431713	0.01725173706942399	0	0	113.11931818181819	488.7491512268038	PPARG
Positive Regulation Of Binding (GO:0051099)	1/91	0.013588540865001511	0.017549020112087376	0	0	110.59444444444445	475.39336307702496	PPARG

Positive Regulation Of Neuron Projection Development (GO:0010976)	1/92	0.013737178126808362	0.017652273892948742	0	0	109.37362637362638	468.95576237961774	EP300
Peptidyl-Lysine Modification (GO:0018205)	1/94	0.014034407867363635	0.017944491651305742	0	0	107.01075268817205	456.53390237907763	EP300
RNA Splicing (GO:0008380)	1/98	0.01462868823763482	0.01852006343385295	0	0	102.57731958762886	433.36565740381525	PPARGC1A
Positive Regulation Of Cold-Induced Thermogenesis (GO:0120162)	1/98	0.01462868823763482	0.01852006343385295	0	0	102.57731958762886	433.36565740381525	PPARGC1A
Cellular Response To Insulin Stimulus (GO:0032869)	1/99	0.014777221017602536	0.018616400987862016	0	0	101.52551020408163	427.8963600882829	PPARG
Response To UV (GO:0009411)	1/100	0.014925738877681147	0.018620946075553663	0	0	100.4949494949495	422.54790984855003	EP300
Regulation Of Organelle Organization (GO:0033043)	1/100	0.014925738877681147	0.018620946075553663	0	0	100.4949494949495	422.54790984855003	EP300
Negative Regulation Of MAPK Cascade (GO:0043409)	1/105	0.015668104356210435	0.019359148170894624	0	0	95.63942307692308	397.4897035431012	PPARG
Regulation Of Neuron Apoptotic Process (GO:0043523)	1/105	0.015668104356210435	0.019359148170894624	0	0	95.63942307692308	397.4897035431012	PPARGC1A
Regulation Of Cellular Response To Stress (GO:0080135)	1/107	0.015964946111592007	0.019631536606120315	0	0	93.8254716981132	388.18973746853214	EP300
Positive Regulation Of Metabolic Process (GO:0009893)	1/108	0.016113344614541864	0.01971351012927109	0	0	92.94392523364486	383.68251411694916	PPARGC1A
Negative Regulation Of Developmental Process (GO:0051093)	1/109	0.016261728200021287	0.01971351012927109	0	0	92.07870370370371	379.26673825723503	PPARG
Positive Regulation Of Cysteine-Type Endopeptidase Activity Involved In Apoptotic Process (GO:0043280)	1/109	0.016261728200021287	0.01971351012927109	0	0	92.07870370370371	379.26673825723503	PPARG
Response To Lipid (GO:0033993)	1/110	0.016410096868260147	0.019793124697412942	0	0	91.22935779816514	374.9397489411658	PPARG
Negative Regulation Of Inflammatory Response (GO:0050728)	1/111	0.016558450622349348	0.019793124697412942	0	0	90.39545454545454	370.69898583844775	PPARG
Negative Regulation Of Transmembrane Receptor Protein Serine/Threonine Kinase Signaling Pathway (GO:0090101)	1/111	0.016558450622349348	0.019793124697412942	0	0	90.39545454545454	370.69898583844775	PPARG
Negative Regulation Of Defense Response (GO:0031348)	1/112	0.016706789460352776	0.01987798560791974	0	0	89.57657657657657	366.54198460977204	PPARG
Regulation Of MAP Kinase Activity (GO:0043405)	1/114	0.017003422399566984	0.020137693809625417	0	0	87.98230088495575	358.4698638821348	PPARG
Cellular Response To Oxidative Stress (GO:0034599)	1/117	0.01744825996477855	0.02056973766489948	0	0	85.69396551724138	346.9333348667141	PPARGC1A
Positive Regulation Of Cell Projection Organization (GO:0031346)	1/118	0.017596509331665674	0.020649784923461548	0	0	84.95726495726495	343.2320001415874	EP300

Regulation Of Protein-Containing Complex Assembly (GO:0043254)	1/119	0.017744743788540594	0.020729087062067875	0	0	84.23305084745763	339.5995214082985	EP300
Fatty Acid Metabolic Process (GO:0006631)	1/122	0.01818935771105458	0.021152330007877952	0	0	82.13223140495867	329.097165436044	PPARGC1A
Positive Regulation Of Intracellular Protein Transport (GO:0090316)	1/126	0.018781967584401812	0.02164558596049895	0	0	79.488	315.953515922278	EP300
Protein-Containing Complex Organization (GO:0043933)	1/126	0.018781967584401812	0.02164558596049895	0	0	79.488	315.953515922278	PPARGC1A
Transmembrane Receptor Protein Serine/Threonine Kinase Signaling Pathway (GO:0007178)	1/130	0.01937433899658254	0.02222859429518622	0	0	77.0077519379845	303.7036203266635	PPARG
Epithelial Cell Differentiation (GO:0030855)	1/132	0.019670435292830718	0.02246800831225553	0	0	75.82442748091603	297.8867663342661	PPARG
Negative Regulation Of Cellular Biosynthetic Process (GO:0031327)	1/137	0.020410415306419013	0.023210074043140207	0	0	73.01838235294117	284.1663654413698	EP300
Regulation Of Cold-Induced Thermogenesis (GO:0120161)	1/146	0.021741440945039152	0.02450679966173273	0	0	68.45517241379311	262.08303164047317	PPARGC1A
Negative Regulation Of Response To External Stimulus (GO:0032102)	1/146	0.021741440945039152	0.02450679966173273	0	0	68.45517241379311	262.08303164047317	PPARG
Negative Regulation Of Protein Phosphorylation (GO:0001933)	1/149	0.02218484812264927	0.024897405971706824	0	0	67.05743243243244	255.37788714760265	PPARG
Positive Regulation Of NF-kappaB Transcription Factor Activity (GO:0051092)	1/152	0.02262812131687884	0.025284465993208095	0	0	65.71523178807946	248.9662194692262	EP300
Epithelium Development (GO:0060429)	1/154	0.022923562351288392	0.02550370356831652	0	0	64.84967320261438	244.84577594566278	PPARG
Mitochondrion Organization (GO:0007005)	1/162	0.024104731206729083	0.0267022237936611	0	0	61.60248447204969	229.49063947550405	PPARGC1A
Positive Regulation Of Cellular Biosynthetic Process (GO:0031328)	1/174	0.025874699270754836	0.02841793894266664	0	0	57.29479768786127	209.3832451405634	PPARGC1A
Regulation Of Neuron Projection Development (GO:0010975)	1/174	0.025874699270754836	0.02841793894266664	0	0	57.29479768786127	209.3832451405634	EP300
RNA Processing (GO:0006396)	1/183	0.027200770128462753	0.02974722520431884	0	0	54.43681318681319	196.2180370936064	PPARGC1A
Negative Regulation Of Macromolecule Metabolic Process (GO:0010605)	1/186	0.02764252620102137	0.030102242515519035	0	0	53.545945945945945	192.14426641897393	PPARG
Regulation Of Angiogenesis (GO:0045765)	1/205	0.03043720969101589	0.03300575059321133	0	0	48.51225490196079	169.40913189219572	PPARG
Negative Regulation Of Cell Differentiation (GO:0045596)	1/207	0.030731075045849994	0.03318439616295566	0	0	48.036407766990294	167.2858735070358	PPARG
mRNA Processing (GO:0006397)	1/214	0.03175913620977989	0.03415103768164616	0	0	46.44131455399061	160.2027902404613	PPARGC1A
Apoptotic Process (GO:0006915)	1/228	0.03381307738313028	0.03620817036443534	0	0	43.54625550660793	147.48714575156393	EP300
Negative Regulation Of Intracellular Signal Transduction (GO:1902532)	1/231	0.034252829422407094	0.03652687618903993	0	0	42.971739130434784	144.98605081167062	PPARG

Positive Regulation Of Developmental Process (GO:0051094)	1/233	0.034545923314361314	0.03668719955285478	0	0	42.59698275862069	143.35868565534332	PPARG
Regulation Of Inflammatory Response (GO:0050727)	1/240	0.03557128496830357	0.03762065941092188	0	0	41.33472803347281	137.9016045245303	PPARG
Regulation Of Autophagy (GO:0010506)	1/241	0.03571770591610492	0.037620698444421986	0	0	41.16041666666667	137.15098449199263	EP300
Cellular Component Assembly (GO:0022607)	1/260	0.0384968891021043	0.040382451017309404	0	0	38.1042471042471	124.11230941104438	PPARGC1A
Negative Regulation Of Signal Transduction (GO:0009968)	1/267	0.03951945113254702	0.04128658106123814	0	0	37.088345864661655	119.83104705578049	PPARG
Positive Regulation Of Cell Differentiation (GO:0045597)	1/283	0.041854012960980874	0.04354850741284245	0	0	34.955673758865245	110.9341935896518	PPARG
Regulation Of Intracellular Signal Transduction (GO:1902531)	1/297	0.043893648920636935	0.04548656359920844	0	0	33.27871621621622	104.02878903876675	EP300
Protein Transport (GO:0015031)	1/313	0.04622111488833046	0.04770613062771457	0	0	31.546474358974358	96.9839114062754	PPARG
Protein-Containing Complex Assembly (GO:0065003)	1/328	0.04839968110370635	0.04975487217461013	0	0	30.07645259938838	91.07938012709077	PPARGC1A
Negative Regulation Of Gene Expression (GO:0010629)	1/336	0.04956022549453898	0.05074493208006581	0	0	29.34626865671642	88.17282076237278	PPARG
Positive Regulation Of Protein Phosphorylation (GO:0001934)	1/377	0.055493208642565066	0.056594264369600085	0	0	26.091755319148938	75.44417045828705	PPARG
Organelle Organization (GO:0006996)	1/418	0.06140145065766503	0.06237222458110637	0	0	23.477218225419666	65.50899423474351	PPARGC1A
Nervous System Development (GO:0007399)	1/433	0.06355683116784583	0.06430750240211172	0	0	22.644675925925927	62.40466879724135	EP300
Regulation Of Nucleic Acid-Templated Transcription (GO:1903506)	1/452	0.06628223971515526	0.06680210041880354	0	0	21.66962305986696	58.807744555934576	PPARGC1A
Negative Regulation Of Nucleic Acid-Templated Transcription (GO:1903507)	1/456	0.06685533531595138	0.06711648896952932	0	0	21.474725274725273	58.09394582203093	PPARG
Negative Regulation Of Apoptotic Process (GO:0043066)	1/482	0.0705747418285925	0.0705747418285925	0	0	20.286902286902286	53.78226102411079	PPARGC1A
