# Supplementary material for: Unveiling the anti-obesity potential of Kemuning (Murraya paniculata): A network pharmacology approach
Source: PLoS One. 2024 Aug 29;19(8):e0305544. doi: 10.1371/journal.pone.0305544 (PMC11361609; doi:10.1371/journal.pone.0305544)
Supplement: S4 Table — (PDF) [file pone.0305544.s004.pdf]

S4 Table. Gene Ontology: Cellular Components of the PPARG, EP300, ad PPARGC1A

Term	Overlap	P-value	Adjusted P-value	Old P-value	Old Adjusted P-value	Odds Ratio	Combined Score	Genes
Histone Acetyltransferase Complex (GO:0000123)	1/27	0.004044678046227062	0.012134034138681185	0	0	384.0576923076923	2116.2935816693675	EP300
Nucleus (GO:0005634)	3/4487	0.011286227025835338	0.01692934053875301	0	0	46539.0	208688.88740652148	EP300;PPARG;PPARGC1A
Intracellular Membrane-Bounded Organelle (GO:0043231)	3/5175	0.017316136866385744	0.017316136866385744	0	0	44475.0	180395.77890880738	EP300;PPARG;PPARGC1A
