# Supplementary material for: Unveiling the anti-obesity potential of Kemuning (Murraya paniculata): A network pharmacology approach
Source: PLoS One. 2024 Aug 29;19(8):e0305544. doi: 10.1371/journal.pone.0305544 (PMC11361609; doi:10.1371/journal.pone.0305544)
Supplement: S5 Table — (PDF) [file pone.0305544.s005.pdf]

S5 Table. Gene Ontology: Molecular Functions of the PPARG, EP300, ad PPARGC1A

Term	Overlap	P-value	Adjusted P-value	Old P-value	Old Adjusted P-value	Odds Ratio	Combined Score	Genes
STAT Family Protein Binding (GO:0097677)	2/9	5.398473530773135E-7	2.4293130888479108E-5	0	0	5711.428571428572	82427.21958358576	EP300;PPARG
RNA Polymerase II-specific DNA-binding Transcription Factor Binding (GO:0061629)	3/228	1.4622004764763584E-6	3.289951072071806E-5	0	0	59316.0	796944.1563059186	EP300;PPARG;PPARGC1A
DNA-binding Transcription Factor Binding (GO:0140297)	3/282	2.7736695264876547E-6	4.160504289731482E-5	0	0	59154.0	756895.5054273832	EP300;PPARG;PPARGC1A
Chromatin DNA Binding (GO:0031490)	2/83	5.0907331593975564E-5	5.657014375953891E-4	0	0	491.75308641975306	4861.226908870382	EP300;PPARGC1A
Transcription Coregulator Binding (GO:0001221)	2/99	7.253017727663914E-5	5.657014375953891E-4	0	0	410.30927835051546	3910.8661052314487	EP300;PPARG
DNA Binding (GO:0003677)	3/846	7.542685834605189E-5	5.657014375953891E-4	0	0	57462.0	545449.2510651937	EP300;PPARG;PPARGC1A
Nuclear Receptor Binding (GO:0016922)	2/115	9.79555079194214E-5	6.297139794819947E-4	0	0	351.929203539823	3248.6574865638645	EP300;PPARGC1A
LBD Domain Binding (GO:0050693)	1/5	7.498314718703743E-4	0.004048892454945414	0	0	2499.125	17982.858997820545	PPARG
Icosatetraenoic Acid Binding (GO:0050543)	1/6	8.997538788767587E-4	0.004048892454945414	0	0	1999.2	14021.167888467728	PPARG
Arachidonic Acid Binding (GO:0050544)	1/6	8.997538788767587E-4	0.004048892454945414	0	0	1999.2	14021.167888467728	PPARG
Icosanoid Binding (GO:0050542)	1/7	0.001049661432299498	0.004294069495770674	0	0	1665.9166666666667	11427.001554688255	PPARG
Peptide N-acetyltransferase Activity (GO:0034212)	1/8	0.0011995541091196305	0.004498327909198614	0	0	1427.857142857143	9603.48923469958	EP300
Prostaglandin Receptor Activity (GO:0004955)	1/9	0.0013494318923464072	0.004671110396583717	0	0	1249.3125	8255.5464452602	PPARG
Nuclear Retinoid X Receptor Binding (GO:0046965)	1/10	0.0014992947695318503	0.004819161759209519	0	0	1110.4444444444443	7220.954198683598	PPARG
Histone H3 Acetyltransferase Activity (GO:0010484)	1/13	0.001948793877001376	0.005846381631004128	0	0	832.7083333333334	5196.553511599695	EP300
Nuclear Retinoic Acid Receptor Binding (GO:0042974)	1/17	0.0025479169262706313	0.007000404310777607	0	0	624.40625	3729.2533063250903	PPARG
R-SMAD Binding (GO:0070412)	1/19	0.002847388777574066	0.007000404310777607	0	0	554.9722222222222	3252.888056951129	PPARG
Actinin Binding (GO:0042805)	1/20	0.0029971022769704784	0.007000404310777607	0	0	525.7368421052631	3054.5885497256336	PPARG
Histone H4 Acetyltransferase Activity (GO:0010485)	1/20	0.0029971022769704784	0.007000404310777607	0	0	525.7368421052631	3054.5885497256336	EP300
N-acetyltransferase Activity (GO:0008080)	1/23	0.0034461530563075866	0.007000404310777607	0	0	453.97727272727275	2574.27663877793	EP300
Alpha-Actinin Binding (GO:0051393)	1/23	0.0034461530563075866	0.007000404310777607	0	0	453.97727272727275	2574.27663877793	PPARG
Nuclear Androgen Receptor Binding (GO:0050681)	1/25	0.0037454454654532016	0.007000404310777607	0	0	416.1041666666667	2324.8633249285726	EP300

Sequence-Specific DNA Binding (GO:0043565)	2/717	0.0037586458894148045	0.007000404310777607	0	0	53.935664335664335	301.16038137568484	PPARG;PPARGC1A
NF-kappaB Binding (GO:0051059)	1/26	0.003895069234369013	0.007000404310777607	0	0	399.44	2216.1106253087632	EP300
Histone Acetyltransferase Activity (GO:0004402)	1/27	0.004044678046227062	0.007000404310777607	0	0	384.0576923076923	2116.2935816693675	EP300
Acetyltransferase Activity (GO:0016407)	1/27	0.004044678046227062	0.007000404310777607	0	0	384.0576923076923	2116.2935816693675	EP300
Transcription Coactivator Binding (GO:0001223)	1/34	0.005091520900981562	0.008485868168302605	0	0	302.4848484848485	1597.1740514070286	EP300
N-acyltransferase Activity (GO:0016410)	1/37	0.005539943461610349	0.008903480563302348	0	0	277.2361111111111	1440.4553417617176	EP300
Tau Protein Binding (GO:0048156)	1/39	0.005838817046122731	0.009060233347431824	0	0	262.61842105263156	1350.7061704673467	EP300
Damaged DNA Binding (GO:0003684)	1/45	0.006735078830116054	0.010102618245174081	0	0	226.73863636363637	1133.7897191126328	EP300
Nuclear Receptor Coactivator Activity (GO:0030374)	1/50	0.007481552373416279	0.010730815623134354	0	0	203.55102040816325	996.4463577649728	PPARGC1A
E-box Binding (GO:0070888)	1/51	0.0076308022208955405	0.010730815623134354	0	0	199.47	972.5284117534744	PPARG
Acyltransferase Activity, Transferring Groups Other Than Amino-Acyl Groups (GO:0016747)	1/64	0.009569689724479127	0.01304957689701699	0	0	158.20634920634922	735.5257596653158	EP300
Transcription Regulatory Region Nucleic Acid Binding (GO:0001067)	1/224	0.033226533718203444	0.043976294627033966	0	0	44.336322869955154	150.9388663798305	PPARG
Ubiquitin Protein Ligase Binding (GO:0031625)	1/271	0.040103446674409765	0.0515615742956697	0	0	36.53148148148148	117.4959480363834	PPARGC1A
Ubiquitin-Like Protein Ligase Binding (GO:0044389)	1/289	0.04272849746075876	0.053410621825948454	0	0	34.217013888888886	107.88245332452784	PPARGC1A
Zinc Ion Binding (GO:0008270)	1/341	0.050285086379747664	0.061157537488882294	0	0	28.90735294117647	86.43433640308764	PPARG
Transition Metal Ion Binding (GO:0046914)	1/456	0.06685533531595138	0.07917079182152137	0	0	21.474725274725273	58.09394582203093	PPARG
Transcription Cis-Regulatory Region Binding (GO:0000976)	1/474	0.06943136371060034	0.08011311197376961	0	0	20.6384778012685	55.05141801530112	PPARG
Double-Stranded DNA Binding (GO:0003690)	1/650	0.09436984171388275	0.1061660719281181	0	0	14.906009244992296	35.186137606119146	PPARG
Sequence-Specific Double-Stranded DNA Binding (GO:1990837)	1/715	0.10346617944663127	0.11356044085605871	0	0	13.503501400560225	30.63283455606341	PPARG
Cis-Regulatory Region Sequence-Specific DNA Binding (GO:0000987)	1/1098	0.15583043935446383	0.16643937355081043	0	0	8.61440291704649	16.01406122722764	PPARG
RNA Polymerase II Cis-Regulatory Region Sequence-Specific DNA Binding (GO:0000978)	1/1122	0.15904206805966328	0.16643937355081043	0	0	8.419268510258698	15.479553698695446	PPARG
RNA Polymerase II Transcription Regulatory Region Sequence-Specific DNA Binding (GO:0000977)	1/1225	0.17273282499163153	0.1766585710141686	0	0	7.668709150326797	13.466324145667045	PPARG
RNA Binding (GO:0003723)	1/1411	0.19707800058638428	0.19707800058638428	0	0	6.591134751773049	10.705028987880905	PPARGC1A
