# Supplementary material for: Unveiling the anti-obesity potential of Kemuning (Murraya paniculata): A network pharmacology approach
Source: PLoS One. 2024 Aug 29;19(8):e0305544. doi: 10.1371/journal.pone.0305544 (PMC11361609; doi:10.1371/journal.pone.0305544)
Supplement: S6 Table — (PDF) [file pone.0305544.s006.pdf]

S6 Table. BioPlanet Pathway of the PPARG, EP300, ad PPARGC1A

Term	Overlap	P-value	Adjusted P-value	Old P-value	Old Adjusted P-value	Odds Ratio	Combined Score	Genes
PPAR-gamma coactivator role in obesity and thermogenesis	3/9	6.299287786297894E-11	6.992209442790662E-9	0	0	59973.0	1408645.7907582622	EP300;PPARG;PPARGC1A
Energy metabolism	3/48	1.2971905887085375E-8	7.199407767332383E-7	0	0	59856.0	1087013.685110418	EP300;PPARG;PPARGC1A
Transcriptional regulation of white adipocyte differentiation	3/77	5.486341776859193E-8	2.0299464574379013E-6	0	0	59769.0	999243.1884115702	EP300;PPARG;PPARGC1A
Lipid metabolism regulation by peroxisome proliferator-activated receptor alpha (PPAR-alpha)	3/112	1.7094585182988947E-7	4.743747388279432E-6	0	0	59664.0	929679.6144138413	EP300;PPARG;PPARGC1A
Fatty acid, triacylglycerol, and ketone body metabolism	3/173	6.360681691871698E-7	1.412071335595517E-5	0	0	59481.0	848672.5344162597	EP300;PPARG;PPARGC1A
Huntington's disease	3/184	7.660804771303328E-7	1.4172488826911157E-5	0	0	59448.0	837145.4644784122	EP300;PPARG;PPARGC1A
CARM1 and regulation of the estrogen receptor	2/26	4.8709953738233275E-6	7.298949560379555E-5	0	0	1664.4166666666667	20359.497943895873	EP300;PPARGC1A
RORA activates circadian expression	2/27	5.2605041876609406E-6	7.298949560379555E-5	0	0	1597.76	19421.226056776162	EP300;PPARGC1A
Developmental biology	3/420	9.195744686606795E-6	1.1341418446815048E-4	0	0	58740.0	681194.2530576688	EP300;PPARG;PPARGC1A
BMAL1-CLOCK/NPAS2 activates circadian expression	2/38	1.0532210921470326E-5	1.1690754122832061E-4	0	0	1108.9444444444443	12709.692443155971	EP300;PPARGC1A
Lipid and lipoprotein metabolism	3/489	1.4528042627164228E-5	1.4660115741956632E-4	0	0	58533.0	652024.2445987765	EP300;PPARG;PPARGC1A
Mechanism of gene regulation by peroxisome proliferators via PPAR-alpha	2/57	2.3896007749647264E-5	2.1872246601272268E-4	0	0	725.1636363636363	7717.045771054736	EP300;PPARGC1A
ATF2 transcription factor network	2/59	2.561614466815671E-5	2.1872246601272268E-4	0	0	699.6491228070175	7396.891853258425	EP300;PPARGC1A
Circadian rhythm	2/62	2.830819651680155E-5	2.2444355809749802E-4	0	0	664.5666666666667	6959.580822872518	EP300;PPARGC1A
Retinoblastoma protein regulation	2/66	3.210631621114883E-5	2.2958343040240556E-4	0	0	622.90625	6444.873216823289	EP300;PPARG
SIDS susceptibility pathways	2/67	3.309310708503143E-5	2.2958343040240556E-4	0	0	613.2923076923076	6326.837237900451	EP300;PPARGC1A
Signaling events mediated by HDAC class I	2/70	3.614288204755832E-5	2.359917592517043E-4	0	0	586.1470588235294	5995.1300122650955	EP300;PPARG
Adipogenesis	2/133	1.3109652020672053E-4	8.084285412747767E-4	0	0	303.2977099236641	2711.3531440096285	PPARG;PPARGC1A
Pathways in cancer	2/325	7.812659338983235E-4	0.004564237824353364	0	0	121.82043343653251	871.5758593009986	EP300;PPARG
PELP1 modulation of estrogen receptor activity	1/7	0.001049661432299498	0.005825620949262213	0	0	1665.9166666666667	11427.001554688255	EP300
Visceral fat deposits and the metabolic syndrome	1/8	0.0011995541091196305	0.0063405002910609035	0	0	1427.857142857143	9603.48923469958	PPARG
Gene expression regulation by hypoxia-inducible factor	1/9	0.0013494318923464072	0.006808497275020509	0	0	1249.3125	8255.5464452602	EP300
Melanocyte development and pigmentation pathway	1/13	0.001948793877001376	0.009317770904440083	0	0	832.7083333333334	5196.553511599695	EP300
TRAF3-dependent IRF activation pathway	1/14	0.0020985970505495682	0.009317770904440083	0	0	768.6153846153846	4739.656184381286	EP300
CARM1 transcriptional regulation by protein methylation	1/14	0.0020985970505495682	0.009317770904440083	0	0	768.6153846153846	4739.656184381286	EP300

Acetylation and deacetylation of RelA in the nucleus	1/16	0.0023981585785517907	0.009506985793544599	0	0	666.0666666666667	4018.4162309154813	EP300
Hypoxia-inducible factor in the cardivascular system	1/16	0.0023981585785517907	0.009506985793544599	0	0	666.0666666666667	4018.4162309154813	EP300
SREBF and miR-33 in cholesterol and lipid homeostasis	1/16	0.0023981585785517907	0.009506985793544599	0	0	666.0666666666667	4018.4162309154813	PPARGC1A
Mitochondrial gene expression	1/19	0.002847388777574066	0.010898626010714528	0	0	554.9722222222222	3252.888056951129	PPARGC1A
Hypoxia and p53 in the cardiovascular system	1/23	0.0034461530563075866	0.011511775977723175	0	0	453.97727272727275	2574.27663877793	EP300
Cellular response to hypoxia	1/25	0.0037454454654532016	0.011511775977723175	0	0	416.1041666666667	2324.8633249285726	EP300
Ghrelin pathway	1/25	0.0037454454654532016	0.011511775977723175	0	0	416.1041666666667	2324.8633249285726	PPARG
RXR/VDR pathway	1/26	0.003895069234369013	0.011511775977723175	0	0	399.44	2216.1106253087632	PPARG
ERBB2 role in signal transduction and oncology	1/26	0.003895069234369013	0.011511775977723175	0	0	399.44	2216.1106253087632	EP300
Estrogen receptor transcription factor targets	1/26	0.003895069234369013	0.011511775977723175	0	0	399.44	2216.1106253087632	PPARGC1A
PGC-1a regulation	1/26	0.003895069234369013	0.011511775977723175	0	0	399.44	2216.1106253087632	PPARGC1A
Fibroblast growth factor 1	1/26	0.003895069234369013	0.011511775977723175	0	0	399.44	2216.1106253087632	PPARG
Cell cycle: G2/M checkpoint	1/27	0.004044678046227062	0.011511775977723175	0	0	384.0576923076923	2116.2935816693675	EP300
Control of gene expression by vitamin D receptor	1/27	0.004044678046227062	0.011511775977723175	0	0	384.0576923076923	2116.2935816693675	EP300
Multi-step regulation of transcription by PITX2	1/28	0.004194271900919973	0.011599279895338214	0	0	369.81481481481484	2024.379431258311	EP300
Thyroid cancer	1/29	0.00434385079686094	0.011599279895338214	0	0	356.5892857142857	1939.4870011141588	PPARG
TRAF6-mediated IRF7 activation	1/30	0.004493414734230119	0.011599279895338214	0	0	344.2758620689655	1860.860040913568	EP300
Retinoic acid receptor-mediated signaling	1/30	0.004493414734230119	0.011599279895338214	0	0	344.2758620689655	1860.860040913568	EP300
NF-kappaB activation by non-typeable Hemophilus influenzae	1/31	0.0046429637132384795	0.011712931185669802	0	0	332.78333333333336	1787.845973802925	EP300
HIF-2-alpha transcription factor network	1/34	0.005091520900981562	0.012119835731597953	0	0	302.4848484848485	1597.1740514070286	EP300
Nuclear receptors in lipid metabolism and toxicity	1/34	0.005091520900981562	0.012119835731597953	0	0	302.4848484848485	1597.1740514070286	PPARG
MEF2D role in T cell apoptosis	1/35	0.005241010046096412	0.012119835731597953	0	0	293.5735294117647	1541.6253665904292	EP300
Interleukin-7 signaling pathway	1/35	0.005241010046096412	0.012119835731597953	0	0	293.5735294117647	1541.6253665904292	EP300
FRA pathway	1/37	0.005539943461610349	0.012382785065711874	0	0	277.2361111111111	1440.4553417617176	EP300
Nuclear receptors	1/38	0.005689387732894645	0.012382785065711874	0	0	269.72972972972974	1394.274144706199	PPARG
p38 alpha/beta MAPK downstream pathway	1/38	0.005689387732894645	0.012382785065711874	0	0	269.72972972972974	1394.274144706199	PPARGC1A
FOXM1 transcription factor network	1/41	0.006137630800512576	0.013101481131863383	0	0	249.4625	1270.5914609940403	EP300
HNF3A pathway	1/44	0.006585739256759462	0.013792774669816987	0	0	232.02325581395348	1165.417705666189	EP300
Regulation of NFAT transcription factors	1/47	0.007033713110919517	0.014195311914764842	0	0	216.8586956521739	1074.9773440238823	PPARG
Regulation of transcription by NOTCH1 intracellular domain	1/47	0.007033713110919517	0.014195311914764842	0	0	216.8586956521739	1074.9773440238823	EP300
HES/HEY pathway	1/48	0.007183007818324503	0.014237747639893212	0	0	212.2340425531915	1047.5951010155554	EP300

FoxO family signaling	1/49	0.007332287573040788	0.014278665273816273	0	0	207.80208333333334	1021.4444344646477	EP300
Nuclear receptor transcription pathway	1/51	0.0076308022208955405	0.014603776664127672	0	0	199.47	972.5284117534744	PPARG
Androgen receptor regulation of biosynthesis and transcription	1/53	0.007929257057990018	0.014730151317210996	0	0	191.77884615384616	927.6718550806611	EP300
Kit receptor signaling pathway	1/54	0.008078462047277996	0.014730151317210996	0	0	188.1509433962264	906.615436737158	EP300
TAp63 pathway	1/55	0.008227652087090826	0.014730151317210996	0	0	184.65740740740742	886.4025678735231	EP300
Calcineurin-dependent NFAT signaling role in lymphocytes	1/55	0.008227652087090826	0.014730151317210996	0	0	184.65740740740742	886.4025678735231	EP300
Pre-NOTCH expression and processing	1/57	0.008525987313688671	0.015021977647927658	0	0	178.04464285714286	848.3179948344085	EP300
Validated nuclear estrogen receptor alpha network	1/64	0.009569689724479127	0.016589134257083436	0	0	158.20634920634922	735.5257596653158	EP300
HIF-1 transcriptional activity in hypoxia	1/66	0.009867755893827221	0.016589134257083436	0	0	153.3230769230769	708.1199963366474	EP300
Adipocytokine signaling pathway	1/67	0.01001676656007227	0.016589134257083436	0	0	150.99242424242425	695.0928600722876	PPARGC1A
PPAR signaling pathway	1/69	0.010314743063972204	0.016589134257083436	0	0	146.53676470588235	670.2856910354703	PPARG
AP-1 transcription factor network	1/70	0.010463708901392551	0.016589134257083436	0	0	144.40579710144928	658.467662524263	EP300
Long-term potentiation	1/70	0.010463708901392551	0.016589134257083436	0	0	144.40579710144928	658.467662524263	EP300
Renal cell carcinoma	1/70	0.010463708901392551	0.016589134257083436	0	0	144.40579710144928	658.467662524263	EP300
Myc repressed pathway	1/73	0.010910516765723988	0.016589134257083436	0	0	138.36805555555554	625.1507650815512	EP300
Signaling by NOTCH1	1/73	0.010910516765723988	0.016589134257083436	0	0	138.36805555555554	625.1507650815512	EP300
Adherens junction cell adhesion	1/74	0.011059422838055624	0.016589134257083436	0	0	136.46575342465752	614.7062292455009	EP300
E2F transcription factor network	1/74	0.011059422838055624	0.016589134257083436	0	0	136.46575342465752	614.7062292455009	EP300
RIG-I/MDA5-mediated induction of interferon-alpha/beta pathways	1/76	0.011357190165993224	0.016808641445669972	0	0	132.81333333333333	594.7253885716618	EP300
p73 transcription factor network	1/79	0.011803729124474183	0.017186595355476977	0	0	127.68589743589743	566.841082547516	EP300
Nuclear beta-catenin signaling and target gene transcription regulation	1/80	0.011952545569943683	0.017186595355476977	0	0	126.0632911392405	558.058364527147	EP300
Regular glucocorticoid receptor pathway	1/82	0.012250133655514035	0.017186595355476977	0	0	122.93827160493827	541.2011251833146	EP300
SMAD2/3 nuclear pathway	1/82	0.012250133655514035	0.017186595355476977	0	0	122.93827160493827	541.2011251833146	EP300
TGF-beta regulation of skeletal system development	1/85	0.012696403776118125	0.017186595355476977	0	0	118.5297619047619	517.552677869987	EP300
Myc active pathway	1/85	0.012696403776118125	0.017186595355476977	0	0	118.5297619047619	517.552677869987	EP300
C-Myb transcription factor network	1/85	0.012696403776118125	0.017186595355476977	0	0	118.5297619047619	517.552677869987	EP300
Androgen receptor signaling, proteolysis, and transcription regulation	1/88	0.013142539507388676	0.017563399912534763	0	0	114.42528735632185	495.67901892070734	EP300
Prostate cancer	1/89	0.013291221555431713	0.017563399912534763	0	0	113.11931818181819	488.7491512268038	EP300
Interferon-gamma signaling pathway	1/97	0.014480140531248308	0.01888121388811006	0	0	103.65104166666667	438.9597968541588	EP300
Wnt signaling pathway and pluripotency	1/98	0.01462868823763482	0.01888121388811006	0	0	102.57731958762886	433.36565740381525	EP300
Melanogenesis	1/101	0.015074241816001008	0.019232653351449563	0	0	99.485	417.31647774293873	EP300
Interleukin-4 signaling pathway	1/104	0.01551966110087719	0.01957593616133373	0	0	96.57281553398059	402.288317326718	EP300

Leptin influence on immune response	1/110	0.016410096868260147	0.02046652530760535	0	0	91.22935779816514	374.9397489411658	PPARG
mTOR signaling pathway	1/113	0.016855113386634184	0.02078797317684883	0	0	88.77232142857143	362.4663724303385	PPARGC1A
p53 activity regulation	1/118	0.017596509331665674	0.02130393239914412	0	0	84.95726495726495	343.2320001415874	EP300
Signaling by NOTCH	1/119	0.017744743788540594	0.02130393239914412	0	0	84.23305084745763	339.5995214082985	EP300
Interleukin-1 regulation of extracellular matrix	1/120	0.0178929633377612	0.02130393239914412	0	0	83.52100840336135	336.03406149176243	PPARG
Notch signaling pathway	1/121	0.01804116797765358	0.02130393239914412	0	0	82.82083333333334	332.53384697347076	EP300
Factors involved in megakaryocyte development and platelet production	1/125	0.01863383747566524	0.021772167997882546	0	0	80.13306451612904	319.15205673213154	EP300
p53 signaling pathway	1/139	0.020706303035718444	0.023941662885049453	0	0	71.95289855072464	278.98420614565964	EP300
Integrated breast cancer pathway	1/152	0.02262812131687884	0.02589403573374795	0	0	65.71523178807946	248.9662194692262	EP300
TGF-beta signaling pathway	1/185	0.027495289038839292	0.031142623299093484	0	0	53.83967391304348	193.48582185240323	EP300
Jak-STAT signaling pathway	1/199	0.029555257303252386	0.033137712733949644	0	0	49.99747474747475	176.0657892808416	EP300
Wnt signaling pathway	1/231	0.034252829422407094	0.038020640658871875	0	0	42.971739130434784	144.98605081167062	EP300
Insulin signaling pathway	1/277	0.04097899608549316	0.0450363224305915	0	0	35.72644927536232	114.13513152336769	PPARGC1A
Oncostatin M	1/311	0.045930388429811154	0.04998306976185331	0	0	31.753225806451614	97.81988678770522	PPARG
Innate immune system	1/319	0.04709293991404629	0.05075064398504017	0	0	30.94182389937107	94.54683296528297	EP300
Generic transcription pathway	1/377	0.055493208642565066	0.05922832845504541	0	0	26.091755319148938	75.44417045828705	PPARG
Cell cycle	1/453	0.0664255356160883	0.0702212805084362	0	0	21.620575221238937	58.627945722979995	EP300
Hemostasis pathway	1/468	0.06857321503628545	0.07180780065120458	0	0	20.910064239828692	56.035904067957496	EP300
TGF-beta regulation of extracellular matrix	1/565	0.08238207421937757	0.08546177792851319	0	0	17.227836879432623	43.00735511516516	PPARG
Gene expression	1/968	0.13829193222803945	0.14213337478992943	0	0	9.83971044467425	19.466768779866715	PPARG
Immune system	1/998	0.1423606329580853	0.1449727546637382	0	0	9.528585757271815	18.574946675648864	EP300
Signal transduction	1/1020	0.1453361930948076	0.1466574312138513	0	0	9.31207065750736	17.960243264604006	EP300
Metabolism	1/1615	0.22322468577466784	0.22322468577466784	0	0	5.694857496902107	8.53987421831147	EP300
