# Supplementary material for: Unveiling the anti-obesity potential of Kemuning (Murraya paniculata): A network pharmacology approach
Source: PLoS One. 2024 Aug 29;19(8):e0305544. doi: 10.1371/journal.pone.0305544 (PMC11361609; doi:10.1371/journal.pone.0305544)
Supplement: S7 Table — (PDF) [file pone.0305544.s007.pdf]

S7 Table. KEGG Pathway of the PPARG, EP300, ad PPARGC1A

Term	Overlap	P-value	Adjusted P-value	Old P-value	Old Adjusted P-value	Odds Ratio	Combined Score	Genes
Huntington disease	3/306	3.5468056810321265E-6	1.3832542156025292E-4	0	0	59082.0	741447.3828744818	EP300;PPARG;PPARGC1A
Longevity regulating pathway	2/102	7.700796942165157E-5	0.0010401276597449484	0	0	397.94	3769.1291576099848	PPARG;PPARGC1A
Glucagon signaling pathway	2/107	8.476791556629031E-5	0.0010401276597449484	0	0	378.8952380952381	3552.367709085901	EP300;PPARGC1A
AMPK signaling pathway	2/120	1.0667975997384087E-4	0.0010401276597449484	0	0	336.93220338983053	3081.473813562663	PPARG;PPARGC1A
Thermogenesis	2/232	3.9886634617831485E-4	0.003111157500190856	0	0	171.88695652173914	1345.3392988875394	PPARG;PPARGC1A
Pathways in cancer	2/531	0.0020735672725505456	0.013478187271578547	0	0	73.60302457466919	454.75517113327186	EP300;PPARG
Thyroid cancer	1/37	0.005539943461610349	0.0308653992861148	0	0	277.2361111111111	1440.4553417617176	PPARG
Notch signaling pathway	1/59	0.008824262742308117	0.031840247878013664	0	0	171.88793103448276	813.0729238663458	EP300
Long-term potentiation	1/67	0.01001676656007227	0.031840247878013664	0	0	150.99242424242425	695.0928600722876	EP300
Adipocytokine signaling pathway	1/69	0.010314743063972204	0.031840247878013664	0	0	146.53676470588235	670.2856910354703	PPARGC1A
Renal cell carcinoma	1/69	0.010314743063972204	0.031840247878013664	0	0	146.53676470588235	670.2856910354703	EP300
Adherens junction	1/71	0.010612659796938678	0.031840247878013664	0	0	142.3357142857143	647.0165481479889	EP300
PPAR signaling pathway	1/74	0.011059422838055624	0.031840247878013664	0	0	136.46575342465752	614.7062292455009	PPARG
TGF-beta signaling pathway	1/94	0.014034407867363635	0.031840247878013664	0	0	107.01075268817205	456.53390237907763	EP300
Prostate cancer	1/97	0.014480140531248308	0.031840247878013664	0	0	103.65104166666667	438.9597968541588	EP300
Melanogenesis	1/101	0.015074241816001008	0.031840247878013664	0	0	99.485	417.31647774293873	EP300
Insulin resistance	1/108	0.016113344614541864	0.031840247878013664	0	0	92.94392523364486	383.68251411694916	PPARGC1A
HIF-1 signaling pathway	1/109	0.016261728200021287	0.031840247878013664	0	0	92.07870370370371	379.26673825723503	EP300
Growth hormone synthesis, secretion and action	1/119	0.017744743788540594	0.031840247878013664	0	0	84.23305084745763	339.5995214082985	EP300
Thyroid hormone signaling pathway	1/121	0.01804116797765358	0.031840247878013664	0	0	82.82083333333334	332.53384697347076	EP300
Cell cycle	1/124	0.018485692459380126	0.031840247878013664	0	0	80.78861788617886	322.40784145177446	EP300
Osteoclast differentiation	1/127	0.01893008279203636	0.031840247878013664	0	0	78.85317460317461	312.81077549345065	PPARG
FoxO signaling pathway	1/131	0.019522394593755113	0.031840247878013664	0	0	76.41153846153846	300.7705652307742	EP300
Insulin signaling pathway	1/137	0.020410415306419013	0.031840247878013664	0	0	73.01838235294117	284.1663654413698	PPARGC1A
Apelin signaling pathway	1/137	0.020410415306419013	0.031840247878013664	0	0	73.01838235294117	284.1663654413698	PPARGC1A
Non-alcoholic fatty liver disease	1/155	0.02307126054382791	0.033210461491046325	0	0	64.42532467532467	242.82984434544923	PPARG
JAK-STAT signaling pathway	1/162	0.024104731206729083	0.033210461491046325	0	0	61.60248447204969	229.49063947550405	EP300
Hepatitis B	1/162	0.024104731206729083	0.033210461491046325	0	0	61.60248447204969	229.49063947550405	EP300
Wnt signaling pathway	1/166	0.02469495854462419	0.033210461491046325	0	0	60.096969696969694	222.42826981279438	EP300
Influenza A	1/172	0.025579853317342374	0.03325380931254509	0	0	57.97076023391813	212.5179210660911	EP300
Tuberculosis	1/180	0.026758880297526048	0.0336643977936618	0	0	55.357541899441344	200.44350827487673	EP300
Transcriptional misregulation in cancer	1/192	0.028525637136025263	0.03388600125487265	0	0	51.84816753926702	184.42144568410632	PPARG
Kaposi sarcoma-associated herpesvirus infection	1/193	0.02867277029258455	0.03388600125487265	0	0	51.575520833333336	183.18631536211723	EP300
Viral carcinogenesis	1/203	0.030143284957031374	0.034249399896274235	0	0	48.99752475247525	171.5791941822581	EP300
Lipid and atherosclerosis	1/215	0.03190594272795973	0.034249399896274235	0	0	46.22196261682243	159.2329507309712	PPARG
cAMP signaling pathway	1/216	0.03205273440608554	0.034249399896274235	0	0	46.0046511627907	158.27314972299234	EP300

Human T-cell leukemia virus 1 infection	1/219	0.03249302041441402	0.034249399896274235	0	0	45.36467889908257	155.45250472614427	EP300
MicroRNAs in cancer	1/310	0.0457850030466385	0.04698987154786584	0	0	31.857605177993527	98.24244098318539	EP300
Human papillomavirus infection	1/331	0.048834995889517306	0.048834995889517306	0	0	29.798484848484847	89.9708064995216	EP300
