# Supplementary material for: Unveiling the anti-obesity potential of Kemuning (Murraya paniculata): A network pharmacology approach
Source: PLoS One. 2024 Aug 29;19(8):e0305544. doi: 10.1371/journal.pone.0305544 (PMC11361609; doi:10.1371/journal.pone.0305544)
Supplement: S8 Table — (PDF) [file pone.0305544.s008.pdf]

S8 Table. Reactome Pathway of the PPARG, EP300, ad PPARGC1A

Term	Overlap	P-value	Adjusted P-value	Old P-value	Old Adjusted P-value	Odds Ratio	Combined Score	Genes
Transcriptional Regulation Of White Adipocyte Differentiation R-HSA-381340	3/84	7.146449429605009E-8	8.40816146110015E-6	0	0	59748.0	983097.4812277551	EP300;PPARG;PPARGC1A
PPARA Activates Gene Expression R-HSA-1989781	3/116	1.901018065610385E-7	8.40816146110015E-6	0	0	59652.0	923156.8193396424	EP300;PPARG;PPARGC1A
Regulation Of Lipid Metabolism By PPARalpha R-HSA-400206	3/118	2.0019432050238453E-7	8.40816146110015E-6	0	0	59646.0	919978.5523969859	EP300;PPARG;PPARGC1A
SUMO E3 Ligases SUMOylate Target Proteins R-HSA-3108232	3/168	5.821921140916228E-7	1.6310130805674243E-5	0	0	59496.0	854152.2625077894	EP300;PPARG;PPARGC1A
SUMOylation R-HSA-2990846	3/174	6.472274129235812E-7	1.6310130805674243E-5	0	0	59478.0	847595.2903982808	EP300;PPARG;PPARGC1A
SUMOylation Of Transcription Cofactors R-HSA-3899300	2/43	1.3526351288293768E-5	2.669144211410416E-4	0	0	973.4634146341464	10913.372597064745	EP300;PPARGC1A
Heme Signaling R-HSA-9707616	2/45	1.4828578952280091E-5	2.669144211410416E-4	0	0	928.0930232558139	10319.423845750922	EP300;PPARGC1A
FOXO-mediated Transcription R-HSA-9614085	2/65	3.113442821719841E-5	4.903672444208749E-4	0	0	632.8253968253969	6566.953388277037	EP300;PPARGC1A
Circadian Clock R-HSA-400253	2/69	3.511139150148894E-5	4.915594810208451E-4	0	0	594.9253731343283	6102.140590249412	EP300;PPARGC1A
Metabolism Of Lipids R-HSA-556833	3/732	4.88321751924866E-5	6.152854074253311E-4	0	0	57804.0	573827.3100673843	EP300;PPARG;PPARGC1A
Developmental Biology R-HSA-1266738	3/1073	1.5400796399405825E-4	0.00176409122393194	0	0	56781.0	498452.3629633787	EP300;PPARG;PPARGC1A
Generic Transcription Pathway R-HSA-212436	3/1190	2.101384709512538E-4	0.002206453944988165	0	0	56430.0	477834.78596696653	EP300;PPARG;PPARGC1A
RNA Polymerase II Transcription R-HSA-73857	3/1312	2.816885170810722E-4	0.0027302117809396226	0	0	56064.0	458306.8655769014	EP300;PPARG;PPARGC1A
Post-translational Protein Modification R-HSA-597592	3/1383	3.299785830973189E-4	0.00296980724787587	0	0	55851.0	447728.58115964354	EP300;PPARG;PPARGC1A
Gene Expression (Transcription) R-HSA-74160	3/1449	3.7954894687373784E-4	0.003188211153739398	0	0	55653.0	438352.3566955732	EP300;PPARG;PPARGC1A
LRR FLII-interacting Protein 1 (LRRFIP1) Activates Type I IFN Production R-HSA-3134973	1/5	7.498314718703743E-4	0.005557574438568657	0	0	2499.125	17982.858997820545	EP300
MECP2 Regulates Transcription Factors R-HSA-9022707	1/5	7.498314718703743E-4	0.005557574438568657	0	0	2499.125	17982.858997820545	PPARG
Metabolism Of Proteins R-HSA-392499	3/1890	8.426758243041559E-4	0.005898730770129092	0	0	54330.0	384598.17040856904	EP300;PPARG;PPARGC1A
Metabolism R-HSA-1430728	3/2049	0.0010738787152023887	0.00712151147976321	0	0	53853.0	368164.8614410649	EP300;PPARG;PPARGC1A
PI5P Regulates TP53 Acetylation R-HSA-6811555	1/9	0.0013494318923464072	0.007871297540042213	0	0	1249.3125	8255.5464452602	EP300
Regulation Of FOXO Transcriptional Activity By Acetylation R-HSA-9617629	1/10	0.0014992947695318503	0.007871297540042213	0	0	1110.4444444444443	7220.954198683598	EP300

Activation Of PPARGC1A (PGC-1alpha) By Phosphorylation R-HSA-2151209	1/10	0.0014992947695318503	0.007871297540042213	0	0	1110.4444444444443	7220.954198683598	PPARGC1A
STAT3 Nuclear Events Downstream Of ALK Signaling R-HSA-9701898	1/10	0.0014992947695318503	0.007871297540042213	0	0	1110.4444444444443	7220.954198683598	EP300
RUNX3 Regulates p14-ARF R-HSA-8951936	1/10	0.0014992947695318503	0.007871297540042213	0	0	1110.4444444444443	7220.954198683598	EP300
Regulation Of Gene Expression By Hypoxia-inducible Factor R-HSA-1234158	1/11	0.0016491427308511233	0.008311679363489661	0	0	999.35	6403.334808352065	EP300
Activation Of TFAP2 (AP-2) Family Of Transcription Factors R-HSA-8866907	1/12	0.0017989757687595922	0.008395220254211432	0	0	908.4545454545455	5741.921288120221	EP300
NOTCH2 Intracellular Domain Regulates Transcription R-HSA-2197563	1/12	0.0017989757687595922	0.008395220254211432	0	0	908.4545454545455	5741.921288120221	EP300
RUNX3 Regulates NOTCH Signaling R-HSA-8941856	1/14	0.0020985970505495682	0.009442749403047675	0	0	768.6153846153846	4739.656184381286	EP300
TRAF3-dependent IRF Activation Pathway R-HSA-918233	1/15	0.0022483852857590027	0.009442749403047675	0	0	713.6785714285714	4351.685756891909	EP300
FOXO-mediated Transcription Of Cell Death Genes R-HSA-9614657	1/16	0.0023981585785517907	0.009442749403047675	0	0	666.0666666666667	4018.4162309154813	EP300
Regulation Of Gene Expression In Late Stage (Branching Morphogenesis) Pancreatic Bud Precursor Cells R-HSA-210744	1/16	0.0023981585785517907	0.009442749403047675	0	0	666.0666666666667	4018.4162309154813	EP300
Polo-like Kinase Mediated Events R-HSA-156711	1/16	0.0023981585785517907	0.009442749403047675	0	0	666.0666666666667	4018.4162309154813	EP300
TP53 Regulates Transcription Of Genes Involved In G2 Cell Cycle Arrest R-HSA-6804114	1/18	0.0026976603266888493	0.009997211798905736	0	0	587.6470588235294	3476.150033460634	EP300
RORA Activates Gene Expression R-HSA-1368082	1/18	0.0026976603266888493	0.009997211798905736	0	0	587.6470588235294	3476.150033460634	EP300
Regulation Of TP53 Activity Thru Methylation R-HSA-6804760	1/19	0.002847388777574066	0.010206348294548116	0	0	554.9722222222222	3252.888056951129	EP300
CD209 (DC-SIGN) Signaling R-HSA-5621575	1/20	0.0029971022769704784	0.010206348294548116	0	0	525.7368421052631	3054.5885497256336	EP300
NOTCH4 Intracellular Domain Regulates Transcription R-HSA-9013695	1/20	0.0029971022769704784	0.010206348294548116	0	0	525.7368421052631	3054.5885497256336	EP300
NOTCH3 Intracellular Domain Regulates Transcription R-HSA-9013508	1/25	0.0037454454654532016	0.011874186595950461	0	0	416.1041666666667	2324.8633249285726	EP300
Cellular Responses To Stress R-HSA-2262752	2/722	0.003810636855356899	0.011874186595950461	0	0	53.547222222222224	298.25582967368484	EP300;PPARGC1A
Attenuation Phase R-HSA-3371568	1/26	0.003895069234369013	0.011874186595950461	0	0	399.44	2216.1106253087632	EP300
Signaling By ALK R-HSA-201556	1/26	0.003895069234369013	0.011874186595950461	0	0	399.44	2216.1106253087632	EP300

Cellular Responses To Stimuli R-HSA-8953897	2/736	0.003958062198650154	0.011874186595950461	0	0	52.48773841961853	290.3622065846168	EP300;PPARGC1A
TRAF6 Mediated IRF7 Activation R-HSA-933541	1/28	0.004194271900919973	0.011898373921836488	0	0	369.81481481481484	2024.379431258311	EP300
FOXO-mediated Transcription Of Oxidative Stress, Metabolic And Neuronal Genes R-HSA-9615017	1/29	0.00434385079686094	0.011898373921836488	0	0	356.5892857142857	1939.4870011141588	PPARGC1A
Regulation Of TP53 Activity Thru Acetylation R-HSA-6804758	1/29	0.00434385079686094	0.011898373921836488	0	0	356.5892857142857	1939.4870011141588	EP300
SUMOylation Of Intracellular Receptors R-HSA-4090294	1/29	0.00434385079686094	0.011898373921836488	0	0	356.5892857142857	1939.4870011141588	PPARG
Metalloprotease DUBs R-HSA-5689901	1/31	0.0046429637132384795	0.012447094209958478	0	0	332.78333333333336	1787.845973802925	EP300
Signaling By NOTCH2 R-HSA-1980145	1/32	0.004792497734793632	0.012580306553833284	0	0	322.03225806451616	1719.8788256956173	EP300
RUNX1 Interacts With Co-Factors Whose Precise Effect On RUNX1 Targets Is Not Known R-HSA-8939243	1/35	0.005241010046096412	0.012815113459952083	0	0	293.5735294117647	1541.6253665904292	EP300
SMAD2/SMAD3:SMAD4 Heterotrimer Regulates Transcription R-HSA-2173796	1/36	0.005390484233154448	0.012815113459952083	0	0	285.1714285714286	1489.484608802673	EP300
Transcriptional Regulation By AP-2 (TFAP2) Family Of Transcription Factors R-HSA-8864260	1/36	0.005390484233154448	0.012815113459952083	0	0	285.1714285714286	1489.484608802673	EP300
HSF1-dependent Transactivation R-HSA-3371571	1/36	0.005390484233154448	0.012815113459952083	0	0	285.1714285714286	1489.484608802673	EP300
NR1H3 And NR1H2 Regulate Gene Expression Linked To Cholesterol Transport And Efflux R-HSA-9029569	1/36	0.005390484233154448	0.012815113459952083	0	0	285.1714285714286	1489.484608802673	EP300
NGF-stimulated Transcription R-HSA-9031628	1/39	0.005838817046122731	0.01362390644095304	0	0	262.61842105263156	1350.7061704673467	EP300
Regulation Of Beta-Cell Development R-HSA-186712	1/41	0.006137630800512576	0.014060754197537903	0	0	249.4625	1270.5914609940403	EP300
NR1H2 And NR1H3-mediated Signaling R-HSA-9024446	1/46	0.0068844034482568265	0.01548990775857786	0	0	221.6888888888889	1103.6774229731402	EP300
NOTCH1 Intracellular Domain Regulates Transcription R-HSA-2122947	1/48	0.007183007818324503	0.015658783630561685	0	0	212.2340425531915	1047.5951010155554	EP300
TP53 Regulates Transcription Of Cell Cycle Genes R-HSA-6791312	1/49	0.007332287573040788	0.015658783630561685	0	0	207.80208333333334	1021.4444344646477	EP300
Signaling By NOTCH3 R-HSA-9012852	1/49	0.007332287573040788	0.015658783630561685	0	0	207.80208333333334	1021.4444344646477	EP300
Transcriptional Activity Of SMAD2/SMAD3:SMAD4 Heterotrimer R-HSA-2173793	1/51	0.007630802220895540 5	0.01581104316869179	0	0	199.47	972.5284117534744	EP300

Formation Of TC-NER Pre-Incision Complex R-HSA-6781823	1/52	0.007780037114753103	0.01581104316869179	0	0	195.54901960784315	949.6240090215041	EP300
Transcriptional Activation Of Mitochondrial Biogenesis R-HSA-2151201	1/52	0.007780037114753103	0.01581104316869179	0	0	195.54901960784315	949.6240090215041	PPARGC1A
Nuclear Receptor Transcription Pathway R-HSA-383280	1/53	0.007929257057990018	0.015858514115980035	0	0	191.77884615384616	927.6718550806611	PPARG
Regulation Of RUNX3 Expression And Activity R-HSA-8941858	1/54	0.008078462047277996	0.015904472155578553	0	0	188.1509433962264	906.615436737158	EP300
Constitutive Signaling By NOTCH1 HD+PEST Domain Mutants R-HSA-2894862	1/58	0.008675132502749212	0.01618918697734142	0	0	174.91228070175438	830.360139497581	EP300
B-WICH Complex Positively Regulates rRNA Expression R-HSA-5250924	1/59	0.008824262742308117	0.01618918697734142	0	0	171.88793103448276	813.0729238663458	EP300
Transcriptional Regulation Of Granulopoiesis R-HSA-9616222	1/59	0.008824262742308117	0.01618918697734142	0	0	171.88793103448276	813.0729238663458	EP300
Formation Of beta-catenin:TCF Transactivating Complex R-HSA-201722	1/60	0.00897337803301339	0.01618918697734142	0	0	168.96610169491527	796.4205513462199	EP300
Transcriptional Regulation By MECP2 R-HSA-8986944	1/60	0.00897337803301339	0.01618918697734142	0	0	168.96610169491527	796.4205513462199	PPARG
Regulation Of PTEN Gene Transcription R-HSA-8943724	1/61	0.009122478376120959	0.01618918697734142	0	0	166.14166666666668	780.3696944503212	PPARG
Nuclear Events (Kinase And Transcription Factor Activation) R-HSA-198725	1/61	0.009122478376120959	0.01618918697734142	0	0	166.14166666666668	780.3696944503212	EP300
Pre-NOTCH Transcription And Translation R-HSA-1912408	1/62	0.00927156377294136	0.01622523660264738	0	0	163.40983606557376	764.8892871354384	EP300
Cytosolic Sensors Of Pathogen-Associated DNA R-HSA-1834949	1/63	0.009420634221166173	0.016260272765300517	0	0	160.76612903225808	749.9503377031319	EP300
Gap-filling DNA Repair Synthesis And Ligation In TC-NER R-HSA-6782210	1/64	0.009569689724479127	0.01629433655789689	0	0	158.20634920634922	735.5257596653158	EP300
Dual Incision In TC-NER R-HSA-6782135	1/65	0.009718730281484593	0.016327466872894113	0	0	155.7265625	721.5902191436588	EP300
RUNX1 Regulates Genes Involved In Megakaryocyte Differentiation And Platelet Function R-HSA-8936459	1/66	0.009867755893827221	0.016359700560818813	0	0	153.3230769230769	708.1199963366474	EP300
Regulation Of RUNX2 Expression And Activity R-HSA-8939902	1/72	0.010761595752488154	0.017418590969937607	0	0	140.32394366197184	635.9160389879967	PPARGC1A
Cellular Response To Hypoxia R-HSA-1234174	1/73	0.010910516765723988	0.017418590969937607	0	0	138.36805555555554	625.1507650815512	EP300
Positive Epigenetic Regulation Of rRNA Expression R-HSA-5250913	1/74	0.011059422838055624	0.017418590969937607	0	0	136.46575342465752	614.7062292455009	EP300

Signaling By NOTCH1 R-HSA-1980143	1/74	0.011059422838055624	0.017418590969937607	0	0	136.46575342465752	614.7062292455009	EP300
Transcription-Coupled Nucleotide Excision Repair (TC-NER) R-HSA-6781827	1/77	0.011506051423331512	0.017692977293652542	0	0	131.05921052631578	585.1639324621284	EP300
Pre-NOTCH Expression And Processing R-HSA-1912422	1/78	0.011654897741056833	0.017692977293652542	0	0	129.35064935064935	575.8728136672166	EP300
Nuclear Events Mediated By NFE2L2 R-HSA-9759194	1/78	0.011654897741056833	0.017692977293652542	0	0	129.35064935064935	575.8728136672166	EP300
DDX58/IFIH1-mediated Induction Of Interferon-Alpha/Beta R-HSA-168928	1/81	0.012101347079866988	0.017938467436038125	0	0	124.48125	549.514822983903	EP300
Signaling By NOTCH4 R-HSA-9013694	1/81	0.012101347079866988	0.017938467436038125	0	0	124.48125	549.514822983903	EP300
Mitochondrial Biogenesis R-HSA-1592230	1/89	0.013291221555431713	0.01945631987488853	0	0	113.11931818181819	488.7491512268038	PPARGC1A
Activation Of HOX Genes During Differentiation R-HSA-5619507	1/91	0.013588540865001511	0.01945631987488853	0	0	110.59444444444445	475.39336307702496	EP300
Signaling By TGF-beta Receptor Complex R-HSA-170834	1/91	0.013588540865001511	0.01945631987488853	0	0	110.59444444444445	475.39336307702496	EP300
Transcriptional Regulation By RUNX3 R-HSA-8878159	1/95	0.014183000347183282	0.02007930386230442	0	0	105.86702127659575	450.5394671189797	EP300
Cellular Response To Heat Stress R-HSA-3371556	1/99	0.014777221017602536	0.020666407676789282	0	0	101.52551020408163	427.8963600882829	EP300
KEAP1-NFE2L2 Pathway R-HSA-9755511	1/100	0.014925738877681147	0.020666407676789282	0	0	100.4949494949495	422.54790984855003	EP300
HATs Acetylate Histones R-HSA-3214847	1/106	0.0158165326939135	0.02166177303731632	0	0	94.72380952380952	392.79117482031205	EP300
Nucleotide Excision Repair R-HSA-5696398	1/108	0.016113344614541864	0.02183098302615349	0	0	92.94392523364486	383.68251411694916	EP300
Signaling By NTRK1 (TRKA) R-HSA-187037	1/114	0.017003422399566984	0.022791821514313193	0	0	87.98230088495575	358.4698638821348	EP300
Epigenetic Regulation Of Gene Expression R-HSA-212165	1/116	0.01729999568673775	0.02281467058526648	0	0	86.44347826086957	350.70542935146375	EP300
Transcriptional Regulation By RUNX2 R-HSA-8878166	1/119	0.017744743788540594	0.02281467058526648	0	0	84.23305084745763	339.5995214082985	PPARGC1A
Estrogen-dependent Gene Expression R-HSA-9018519	1/119	0.017744743788540594	0.02281467058526648	0	0	84.23305084745763	339.5995214082985	EP300
Signaling By TGFB Family Members R-HSA-9006936	1/119	0.017744743788540594	0.02281467058526648	0	0	84.23305084745763	339.5995214082985	EP300
Signaling By NTRKs R-HSA-166520	1/132	0.019670435292830718	0.02503509946360273	0	0	75.82442748091603	297.8867663342661	EP300
PTEN Regulation R-HSA-6807070	1/139	0.020706303035718444	0.02608994182500524	0	0	71.95289855072464	278.98420614565964	PPARG
C-type Lectin Receptors (CLRs) R-HSA-5621481	1/141	0.0210021311855888	0.026200678508754346	0	0	70.91785714285714	273.96499801043336	EP300
Regulation Of TP53 Activity R-HSA-5633007	1/157	0.023366612280172203	0.028864638699036254	0	0	63.592948717948715	238.88354786430114	EP300

G2/M Transition R-HSA-69275	1/182	0.027053488384102493	0.03277634169612417	0	0	54.74033149171271	197.60927511890054	EP300
Cellular Response To Chemical Stress R-HSA-9711123	1/182	0.027053488384102493	0.03277634169612417	0	0	54.74033149171271	197.60927511890054	EP300
Mitotic G2-G2/M Phases R-HSA-453274	1/184	0.027348037017313847	0.032817644420776615	0	0	54.13661202185792	194.84365013551567	EP300
ESR-mediated Signaling R-HSA-8939211	1/188	0.027936955947393486	0.033208079711052635	0	0	52.967914438502675	189.50886279946315	EP300
TCF Dependent Signaling In Response To WNT R-HSA-201681	1/198	0.02940821326513827	0.034630232443060016	0	0	50.253807106598984	177.21911005498902	EP300
Signaling By NOTCH R-HSA-157118	1/203	0.030143284957031374	0.03501442291910353	0	0	48.99752475247525	171.5791941822581	EP300
Transcriptional Regulation By RUNX1 R-HSA-8878171	1/204	0.030290254747478457	0.03501442291910353	0	0	48.75369458128079	170.48822031946239	EP300
Chromatin Modifying Enzymes R-HSA-3247509	1/238	0.03527839860951162	0.0404098020436224	0	0	41.687763713080166	139.4240770668133	EP300
Signaling By Nuclear Receptors R-HSA-9006931	1/260	0.0384968891021043	0.04369917141319947	0	0	38.1042471042471	124.11230941104438	EP300
PIP3 Activates AKT Signaling R-HSA-1257604	1/268	0.039665472216447326	0.04462365624350324	0	0	36.947565543071164	119.23992455638721	PPARG
Organelle Biogenesis And Maintenance R-HSA-1852241	1/275	0.04068720546211637	0.04536803440908551	0	0	35.99087591240876	115.23708364717754	PPARGC1A
Deubiquitination R-HSA-5688426	1/279	0.04127072754305296	0.04561501465284801	0	0	35.46582733812949	113.05093529250024	EP300
Signal Transduction R-HSA-162582	2/2465	0.04181476629054708	0.04581443958790376	0	0	14.237921234267153	45.1983627022705	EP300;PPARG
Signaling By WNT R-HSA-195721	1/294	0.04345682797621647	0.04720310625002824	0	0	33.62457337883959	105.44623488285703	EP300
Intracellular Signaling By Second Messengers R-HSA-9006925	1/306	0.04520331383416838	0.048680491821412104	0	0	32.281967213114754	99.96385156607936	PPARG
DNA Repair R-HSA-73894	1/310	0.0457850030466385	0.04888907104980044	0	0	31.857605177993527	98.24244098318539	EP300
Transcriptional Regulation By TP53 R-HSA-3700989	1/354	0.052167999745345556	0.055236705612718824	0	0	27.824362606232295	82.17330064888138	EP300
Diseases Of Signal Transduction By Growth Factor Receptors And Second Messengers R-HSA-5663202	1/424	0.06226399928230898	0.06537719924642443	0	0	23.1371158392435	64.23723782008254	EP300
Signaling By Receptor Tyrosine Kinases R-HSA-9006934	1/496	0.07257339926421082	0.07557230006025259	0	0	19.6989898989899	51.67353981120037	EP300
Cell Cycle, Mitotic R-HSA-69278	1/523	0.07641985751283839	0.07892542661161998	0	0	18.654214559386972	47.96954967582487	EP300
Cell Cycle R-HSA-1640170	1/654	0.09493138491556997	0.09724678454765705	0	0	14.811638591117918	34.87549774058632	EP300
Innate Immune System R-HSA-168249	1/1035	0.1473610330296143	0.14973782388493068	0	0	9.16972920696325	17.55883658128121	EP300
Disease R-HSA-1643685	1/1736	0.23846170433496017	0.24036939796963985	0	0	5.262824207492796	7.544503487867963	EP300
Immune System R-HSA-168256	1/1943	0.2640640064234834	0.2640640064234834	0	0	4.648558187435634	6.189851603058266	EP300
