# Supplementary material for: Unveiling the anti-obesity potential of Kemuning (Murraya paniculata): A network pharmacology approach
Source: PLoS One. 2024 Aug 29;19(8):e0305544. doi: 10.1371/journal.pone.0305544 (PMC11361609; doi:10.1371/journal.pone.0305544)
Supplement: S9 Table — (PDF) [file pone.0305544.s009.pdf]

S9 Table. Dataset of targets from OMIM and STITCH, and *Murraya paniculata* compounds from IJAH Analytics

Targets from OMIM								
**No**	**MIM ID**	**Gene Symbol**	**No**	**MIM ID**	**Gene Symbol**	**No**	**MIM ID**	**Gene Symbol**
1	615921	PERM1	41	610372	SLC2A12	81	601413	DIO2
2	616593	C1QTNF12	42	143054	HIVEP2	82	616855	COX8C
3	605557	PRDM16	43	619393	KBTBD2	83	616036	MIR494
4	608507	MFN2	44	619734	EPDR1	84	611761	LMF1
5	602284	BMP8A	45	179550	RALA	85	602402	FOXC2
6	614397	MFSD2A	46	611325	TBRG4	86	614245	ACSF3
7	606803	ACOT11	47	616550	TMEM120A	87	612211	TRARG1
8	610824	SLC25A44	48	602186	VGF	88	184756	SREBF1
9	611673	TRMT1L	49	176912	PRKAR2B	89	604694	AKAP10
10	600262	PTGS2	50	164160	LEP	90	619894	ABHD15
11	617124	PM20D1	51	601500	SMO	91	602408	NR1D1
12	613220	TMEM18	52	163729	NOS3	92	616241	METRNL
13	617845	MFSD2B	53	109691	ADRB3	93	604440	CIDEA
14	602691	NCOA1	54	601993	NCOA2	94	606211	SIRT6
15	600291	ADCY3	55	612757	GPIHBP1	95	147670	INSR
16	136435	FSHR	56	609312	DBH	96	605565	RETN
17	610736	ANKRD23	57	607001	EHMT1	97	605910	ANGPTL4
18	617529	FASTKD1	58	601299	BMPR1A	98	616223	ANGPTL8
19	609576	ACADL	59	611815	ELOVL3	99	116897	CEBPA
20	610147	GPBAR1	60	607035	SUFU	100	190180	TGFB1
21	610268	MOGAT1	61	602714	ADAM12	101	151750	LIPE
22	612120	CIDEC	62	604481	SIRT3	102	609436	FGF21
23	605353	GHRL	63	612793	PTDSS2	103	618833	RALGAPB
24	601487	PPARG	64	113505	BDNF	104	612029	FITM2
25	608298	CCDC80	65	136530	FSHB	105	614168	PCK1
26	611909	FNDC3B	66	606158	BSCL2	106	139320	GNAS
27	610280	OSTN	67	600526	MARK2	107	604699	ARFRP1
28	617869	NKX1-1	68	601398	VEGFB	108	609196	MRAP
29	616238	ZBTB49	69	102574	ACTN3	109	615588	SMDT1
30	604517	PPARGC1A	70	601693	UCP2	110	609567	PNPLA3
31	610958	GPAT3	71	602044	UCP3	111	313700	AR
32	608468	NOCT	72	608973	SIK2	112	300996	YIPF6
33	113730	UCP1	73	604773	ACAD8	113	300827	FGF16
34	600983	NR3C2	74	611324	CLSTN3	114	300242	SLC25A14
35	600857	SDHA	75	618044	C2CD5			
36	147780	IL4	76	602113	KMT2D			
37	605166	HDAC3	77	159990	MYF5			
38	608886	PPARGC1B	78	616551	TMEM120B			
39	601900	IRF4	79	604824	KL			
40	615652	ACOT13	80	614041	RB1			

Targets from STITCH					
No	Gene Symbol	No	Gene Symbol	No	Gene Symbol
1	PMF1	23	CASP1	45	BDKRB2
2	ENSG00000260238	24	CYP1B1	46	ADRA1A
3	UGT1A10	25	NOS1	47	ADRA1D
4	UGT1A8	26	NOS2	48	ADRA1B
5	UGT1A3	27	PPARG	49	CYP2D6
6	UGT1A7	28	NOS3	50	CYP2C19
7	UGT1A9	29	MTRR	51	CYP3A4
8	UGT1A1	30	POR	52	AURKB
9	UGT1A6	31	IPP	53	F2
10	MITF	32	CYP1A1	54	WNT1
11	CASP3	33	CYP1A2	55	MMP9
12	UGT2B15	34	GOT1	56	WNT3A
13	VR1	35	B2M	57	HGF
14	TRPV1	36	GFAP	58	NFE2L2
15	CACNA1G	37	GOT1L1	59	BGLAP
16	TRPA1	38	GOT2	60	CCL11
17	UGT2B17	39	ACAD10	61	WNT3
18	CACNA1H	40	CES1	62	CDK2
19	ALOX5	41	CES4A	63	DDC
20	MAOA	42	CES2	64	CER1
21	TMPRSS11D	43	CES5A		
22	FIP1L1	44	CES3		

***Murraya paniculata* compounds from IJAH Analytics**	
**No.**	**Compound**
1	Mahanimbinine
2	Isomurralonginol acetate
3	Murranganone
4	Mexolide
5	5,6,7,3',4',5'-Hexamethoxyflavone
6	erythro-Murrangatin
7	(+)-Murrayazoline
8	Murrayone
9	Eugenol
10	Murraxonin
11	Peroxymurraol
12	Murrangatin diacetate
13	Murralongin
14	8-Hydroxy-3,5,7,3',4',5'-hexamethoxyflavone
15	5-Hydroxy-3,3',4',5',7,8-hexamethoxyflavone
16	Murraculatin
17	Murracarpin
18	Scopoletin
19	Hexa-O-methylmyricitin
20	Yuehchukene
21	Imperatorin

22	D-Limonene
23	Skimmianine
24	Omphamurrayin
25	Demethylnobiletin
26	Murrayacarpin A
27	Murralonginol isovalerate
28	8-[(2R)-2,3-dihydroxy-3-methylbutyl]-5,7-dimethoxychromen-2-one
29	Omphalocarpin
30	3-Hydroxy-5,7,3',4',5'-pentamethoxyflavone
31	,5,8-Trihydroxy-6,7,3',4'-tetramethoxyflavone
32	beta-Pinene
33	Gardenin A
34	Sinensetin
35	Semi-alpha-carotenone
36	Gardenin C
37	Vomifoliol
38	Gardenin E
39	Murraxocin
40	Paniculidine B
41	(-)-Minumicrolin
42	Hibiscetin heptamethyl ether
43	Methyl salicylate
44	Tamynine
45	Meranzin
46	Microminutin
47	threo-Murrangatin
48	8-(1-Chloro-2-hydroxy-3-methylbut-3-enyl)-7-methoxycoumarin
49	Murrayanine
50	(+)-Mahanimbicine
51	Isomexoticin
52	5,3',5'-Trihydroxy-6,7,4'-trimethoxyflavone
53	8-(Hydroxymethyl)-5,7-dimethoxychromen-2-one
54	8-[(2S)-2,3-dihydroxy-3-methylbutyl]-7-methoxychromen-2-one
55	Murrayanone
56	Scopolin
57	Paniculatin
58	Osthole
59	Murrayatin
60	Paniculidine C
61	(-)-Citronellol
62	Geraniol
63	Isomurralonginol isovalerate
64	3',4',5',5,7-Pentamethoxyflavone
65	Gossypetin hexamethyl ether
66	Gossypetin 3,7,8,3',4'-pentamethyl ether
67	Bannamurpanisin

68	Umuhengerin
69	3',4',5',3,5,6,7-Heptamethoxyflavone
70	5-Hydroxy-3,6,7,3',4',5'-hexamethoxyflavone
71	4'-Hydroxy-3,5,6,7,3',5'-hexamethoxyflavone
72	alpha-Pinene
73	Noracronycine
74	Paniculonol isovalerate
75	5-Hydroxy-2-(3-hydroxy-4,5-dimethoxyphenyl)-6,7-dimethoxychromen-4-one
76	Murraol
