# Supplementary material for: Unveiling the anti-obesity potential of Kemuning (Murraya paniculata): A network pharmacology approach
Source: PLoS One. 2024 Aug 29;19(8):e0305544. doi: 10.1371/journal.pone.0305544 (PMC11361609; doi:10.1371/journal.pone.0305544)
Supplement: S1 File — (A) Native ligand BRL, (B) (1R,9S)-5-[(E)-2-(4-chlorophenyl)ethenyl]-11-(pyrimidine-5-carbonyl)-7,11-diazatricyclo [7.3.1.02,7]trideca-2,4-dien-6-one, (C) 4-Aminobenzoic acid, (D) alpha-Lapachone, (E) DL-Tryptophan, (F) Hainanmurpanin, (G) L-Phenylalanine, (H) Murralongin, (I) Murrangatin, (J) Murraol, and (K) trans-3-Indoleacrylic acid. (PDF) [file pone.0305544.s010.pdf]

S1 File. Binding results of molecular docking between PPARG (7AWC) and ligands. (A) Native ligand BRL, (B) (1R,9S)-5-[(E)-2-(4-chlorophenyl)ethenyl]-11-(pyrimidine-5-carbonyl)-7,11-diazatricyclo[7.3.1.02,7]trideca-2,4-dien-6-one, (C) 4-Aminobenzoic acid, (D) alpha-Lapachone, (E) DL-Tryptophan, (F) Hainanmurpanin, (G) L-Phenylalanine, (H) Murralongin, (I) Murrangatin, (J) Murraol, (K) trans-3-Indoleacrylic acid

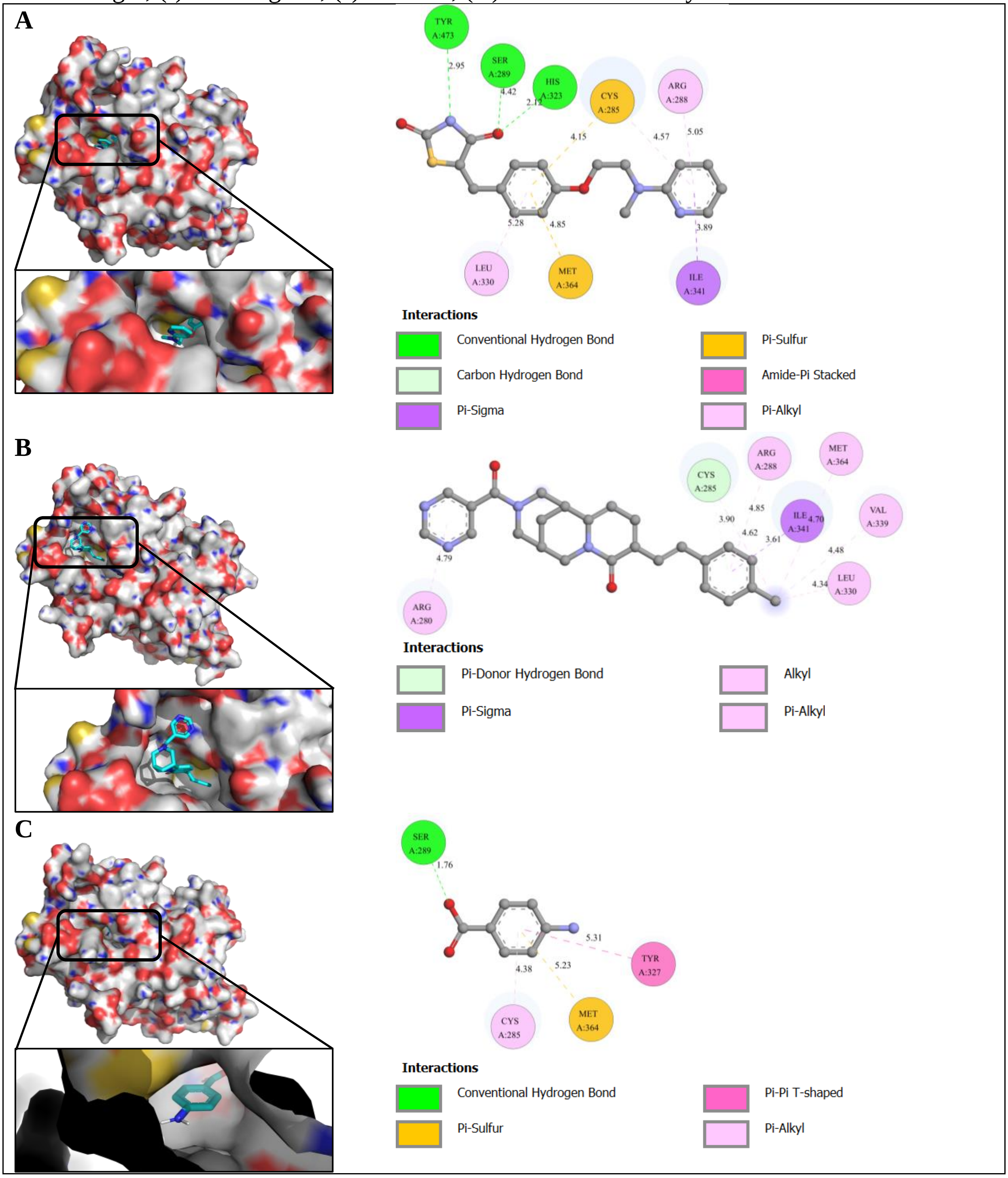

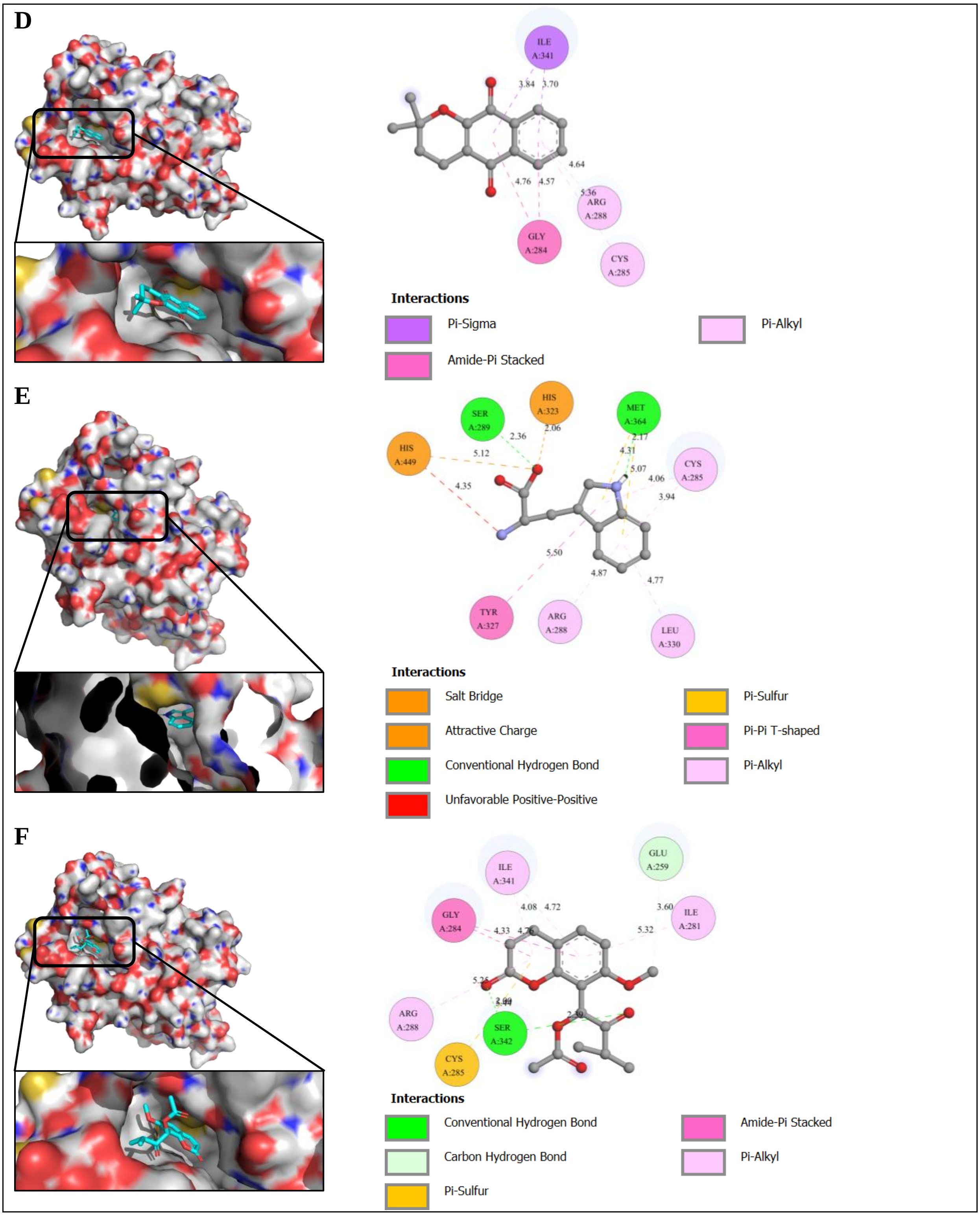
D
ILE A:341
3.84
3.70
4.64
4.76
4.57
5.36
ARG A:288
GLY A:284
CYS A:285
Interactions
Pi-Sigma
Pi-Alkyl
Amide-Pi Stacked
E
HIS A:323
SER A:289
MET A:364
HIS A:449
2.36
2.06
2.17
5.12
4.31
5.07
CYS A:285
4.06
4.35
3.94
5.50
4.87
4.77
TYR A:327
ARG A:288
LEU A:330
Interactions
Salt Bridge
Pi-Sulfur
Attractive Charge
Pi-Pi T-shaped
Conventional Hydrogen Bond
Pi-Alkyl
Unfavorable Positive-Positive
F
GLU A:259
ILE A:341
4.08
4.72
3.60
GLY A:284
ILE A:281
4.33
5.32
ARG A:288
SER A:342
CYS A:285
Interactions
Conventional Hydrogen Bond
Amide-Pi Stacked
Carbon Hydrogen Bond
Pi-Alkyl
Pi-Sulfur

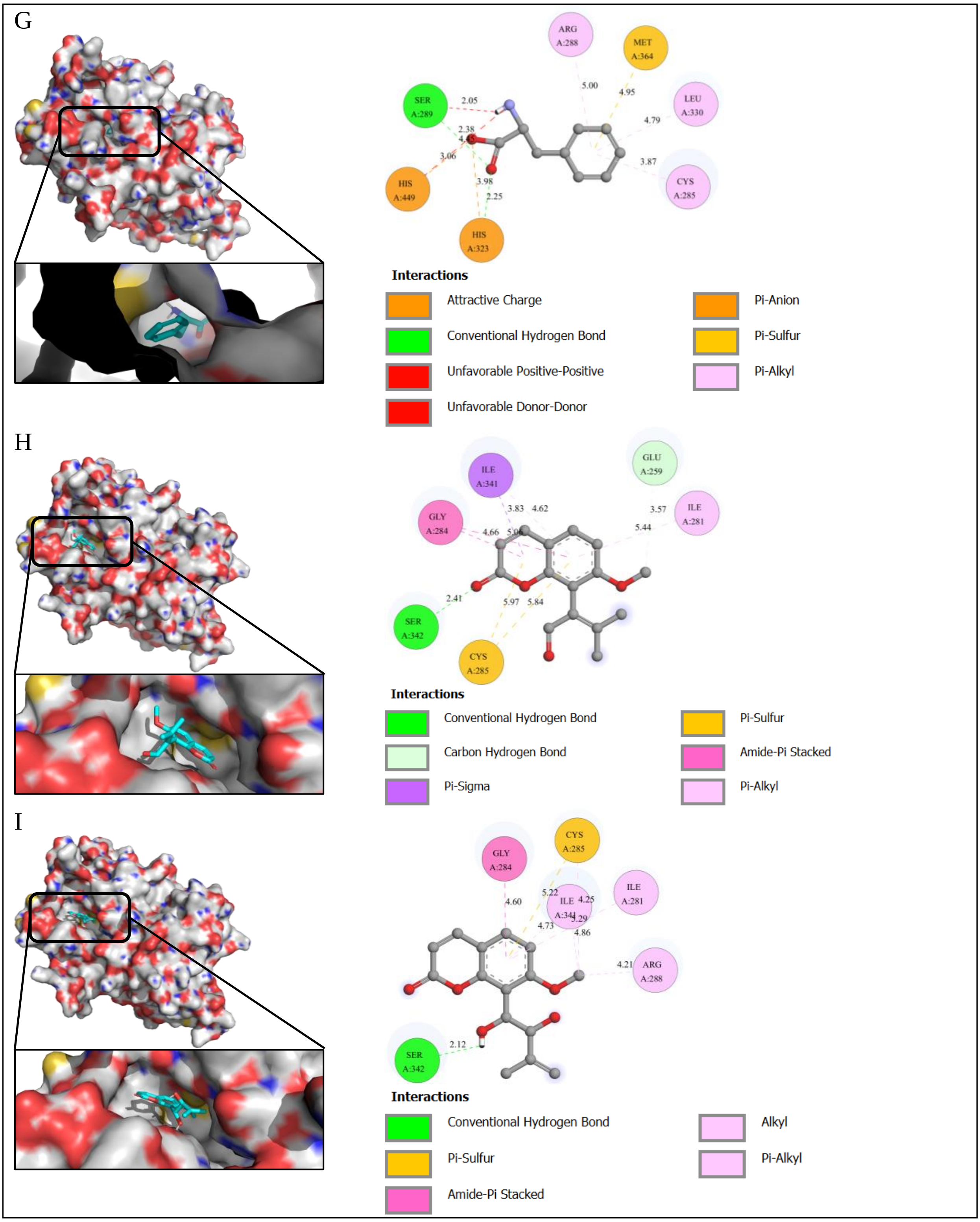
G
ARG A:288
MET A:364
SER A:289
LEU A:330
HIS A:449
CYS A:285
HIS A:323
2.05
2.38
3.06
3.98
2.25
5.00
4.95
4.79
3.87
Interactions
Attractive Charge
Conventional Hydrogen Bond
Unfavorable Positive-Positive
Unfavorable Donor-Donor
Pi-Anion
Pi-Sulfur
Pi-Alkyl
H
ILE A:341
GLU A:259
GLY A:284
ILE A:281
SER A:342
CYS A:285
3.83
4.62
3.57
5.44
4.66
2.41
5.97
5.84
Interactions
Conventional Hydrogen Bond
Carbon Hydrogen Bond
Pi-Sigma
Pi-Sulfur
Amide-Pi Stacked
Pi-Alkyl
I
CYS A:285
GLY A:284
ILE A:281
ARG A:288
SER A:342
4.60
5.22
4.25
4.73
4.86
4.21
2.12
Interactions
Conventional Hydrogen Bond
Pi-Sulfur
Amide-Pi Stacked
Alkyl
Pi-Alkyl

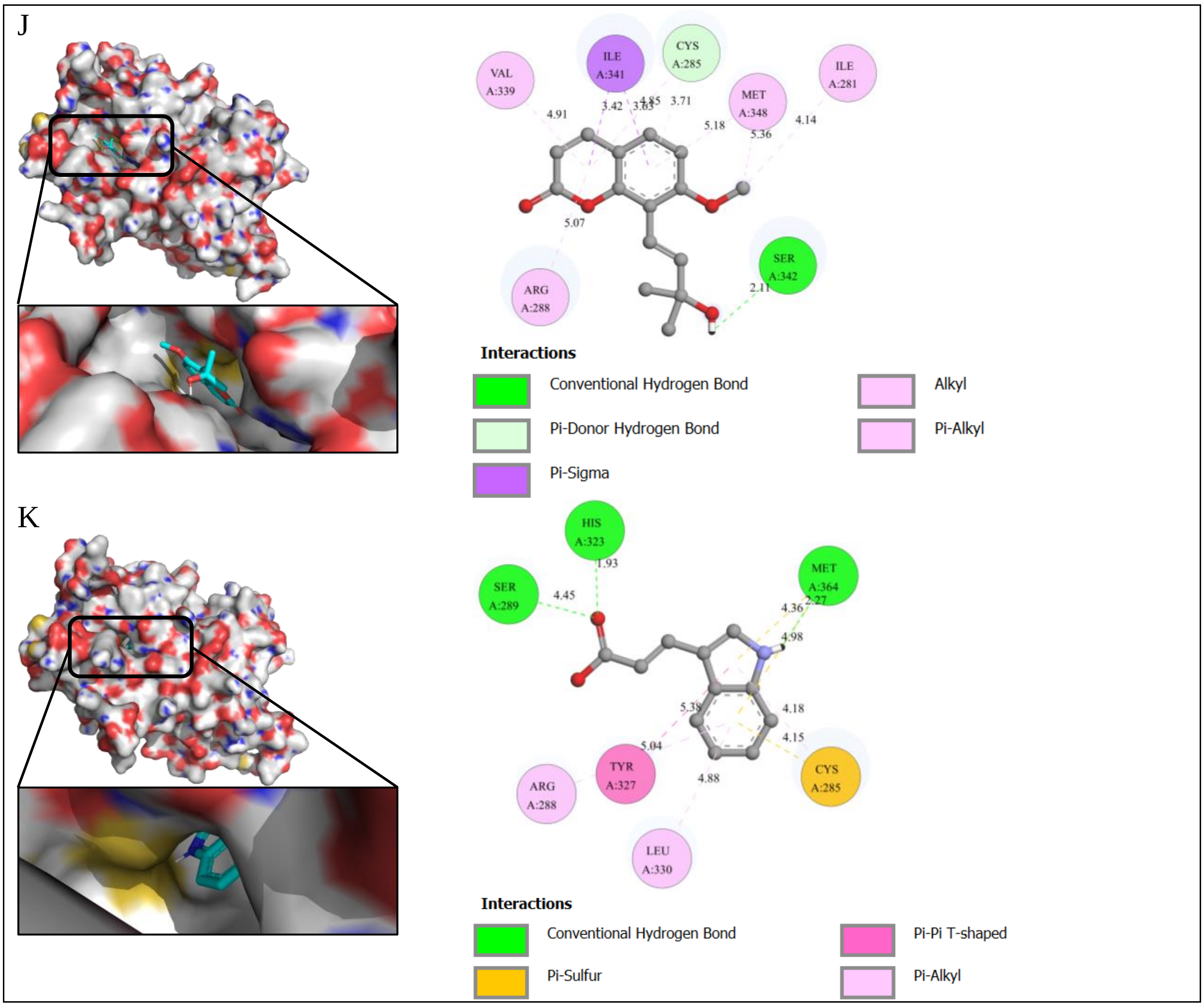
J
VAL A:339
ILE A:341
CYS A:285
MET A:348
ILE A:281
SER A:342
ARG A:288
Interactions
Conventional Hydrogen Bond
Pi-Donor Hydrogen Bond
Pi-Sigma
Alkyl
Pi-Alkyl
K
HIS A:323
SER A:289
MET A:364
TYR A:327
ARG A:288
CYS A:285
LEU A:330
Interactions
Conventional Hydrogen Bond
Pi-Sulfur
Pi-Pi T-shaped
Pi-Alkyl
