# Supplementary material for: Unveiling the anti-obesity potential of Kemuning (Murraya paniculata): A network pharmacology approach
Source: PLoS One. 2024 Aug 29;19(8):e0305544. doi: 10.1371/journal.pone.0305544 (PMC11361609; doi:10.1371/journal.pone.0305544)
Supplement: S2 File — (A) Native ligand 99E, (B) (1R,9S)-5-[(E)-2-(4-chlorophenyl)ethenyl]-11-(pyrimidine-5-carbonyl)-7,11-diazatricyclo [7.3.1.02,7]trideca-2,4-dien-6-one, (C) 4-Aminobenzoic acid, (D) alpha-Lapachone, (E) DL-Tryptophan, (F) Hainanmurpanin, (G) L-Phenylalanine, (H) Murralongin, (I) Murrangatin, (J) Murraol, and (K) trans-3-Indoleacrylic acid. (PDF) [file pone.0305544.s011.pdf]

S2 File. Binding results of molecular docking between EP300 (5NU5) and ligands. (**A**) Native ligand 99E, (**B**) (1R,9S)-5-[(E)-2-(4-chlorophenyl)ethenyl]-11-(pyrimidine-5-carbonyl)-7,11-diazatricyclo[7.3.1.02,7]trideca-2,4-dien-6-one, (**C**) 4-Aminobenzoic acid, (**D**) alpha-Lapachone, (**E**) DL-Tryptophan, (**F**) Hainanmurpanin, (**G**) L-Phenylalanine, (**H**) Murralongin, (**I**) Murrangatin, (**J**) Murraol, (**K**) trans-3-Indoleacrylic acid

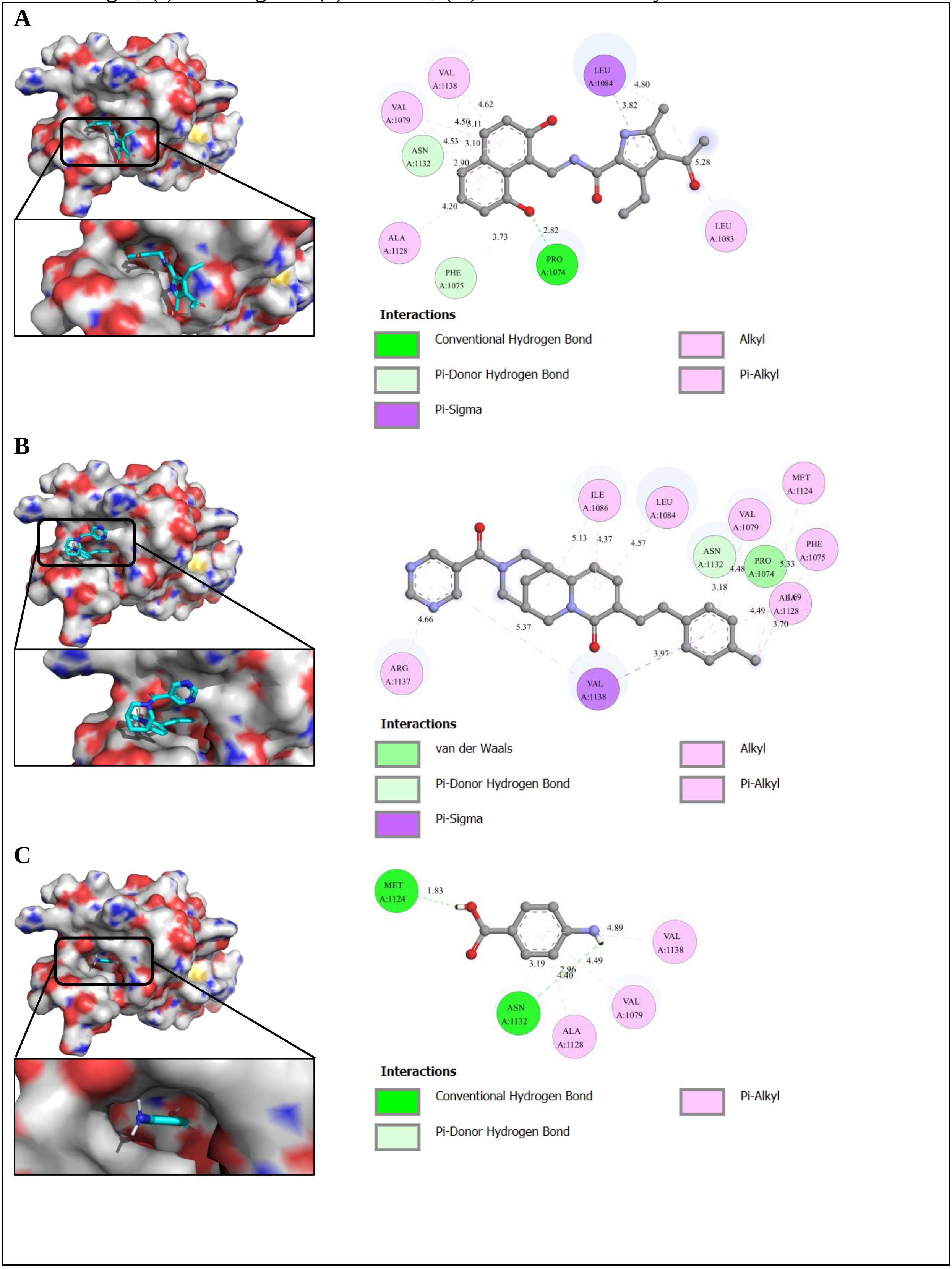

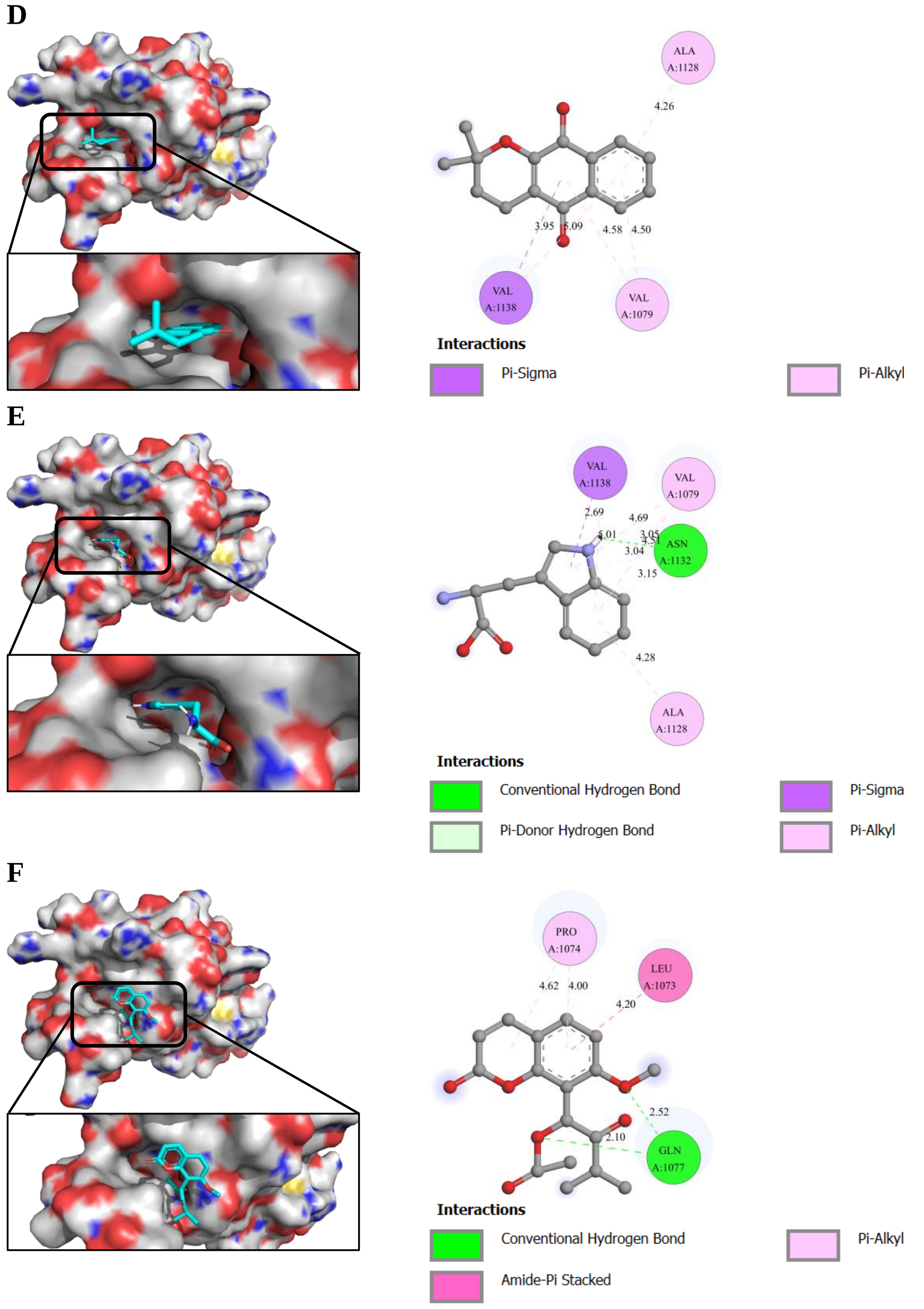
D
ALA A:1128
4.26
3.95
5.09
4.58
4.50
VAL A:1138
VAL A:1079
Interactions
Pi-Sigma
Pi-Alkyl
E
VAL A:1138
VAL A:1079
2.69
4.69
5.01
3.05
4.51
3.04
ASN A:1132
3.15
4.28
ALA A:1128
Interactions
Conventional Hydrogen Bond
Pi-Sigma
Pi-Donor Hydrogen Bond
Pi-Alkyl
F
PRO A:1074
LEU A:1073
4.62
4.00
4.20
2.52
2.10
GLN A:1077
Interactions
Conventional Hydrogen Bond
Pi-Alkyl
Amide-Pi Stacked

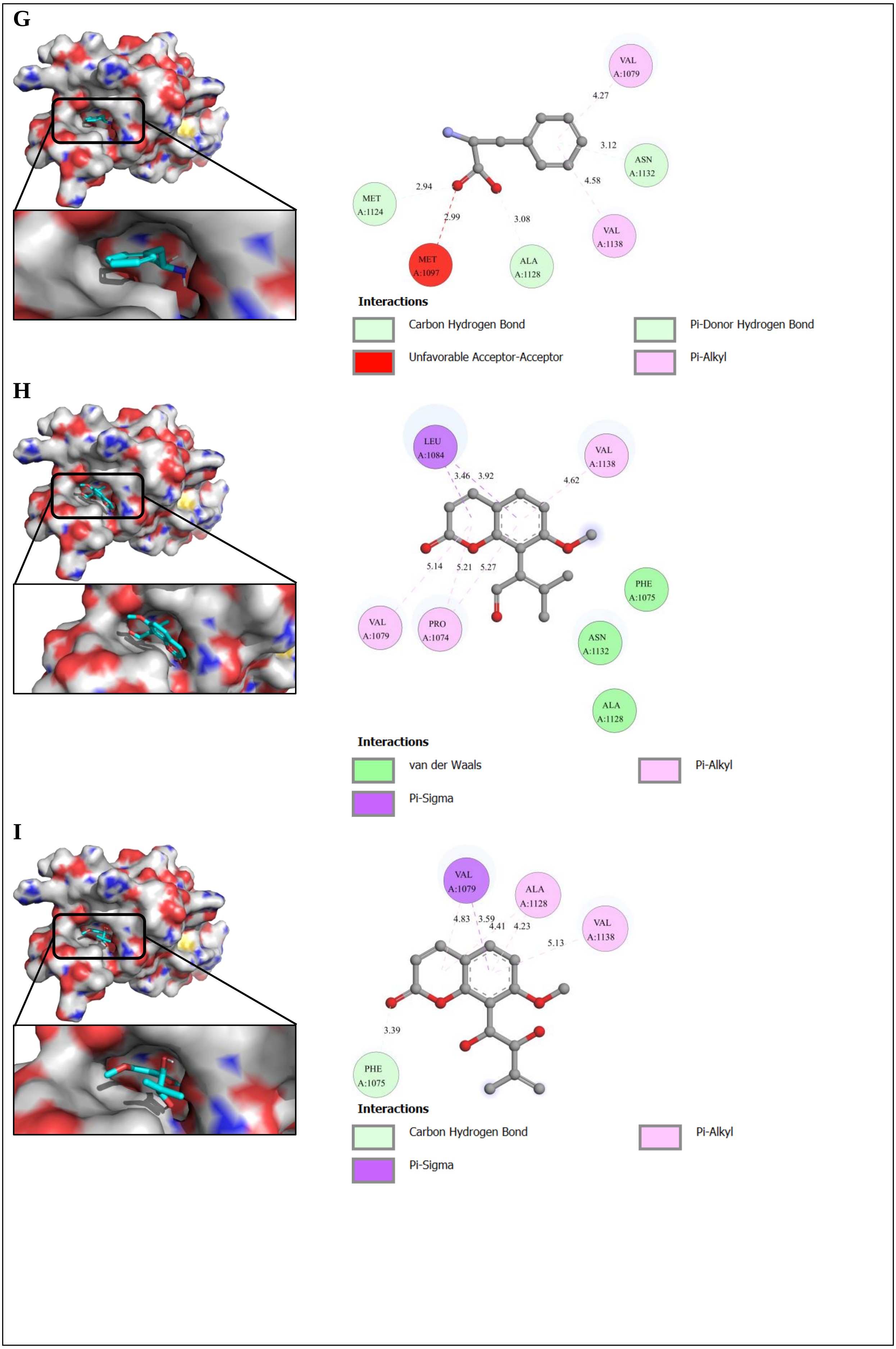
G
VAL A:1079
4.27
3.12
ASN A:1132
4.58
2.94
MET A:1124
2.99
3.08
VAL A:1138
MET A:1097
ALA A:1128
Interactions
Carbon Hydrogen Bond
Pi-Donor Hydrogen Bond
Unfavorable Acceptor-Acceptor
Pi-Alkyl
H
LEU A:1084
VAL A:1138
3.46
3.92
4.62
5.14
5.21
5.27
PHE A:1075
VAL A:1079
PRO A:1074
ASN A:1132
ALA A:1128
Interactions
van der Waals
Pi-Alkyl
Pi-Sigma
I
VAL A:1079
ALA A:1128
4.83
3.59
4.41
4.23
VAL A:1138
5.13
3.39
PHE A:1075
Interactions
Carbon Hydrogen Bond
Pi-Alkyl
Pi-Sigma

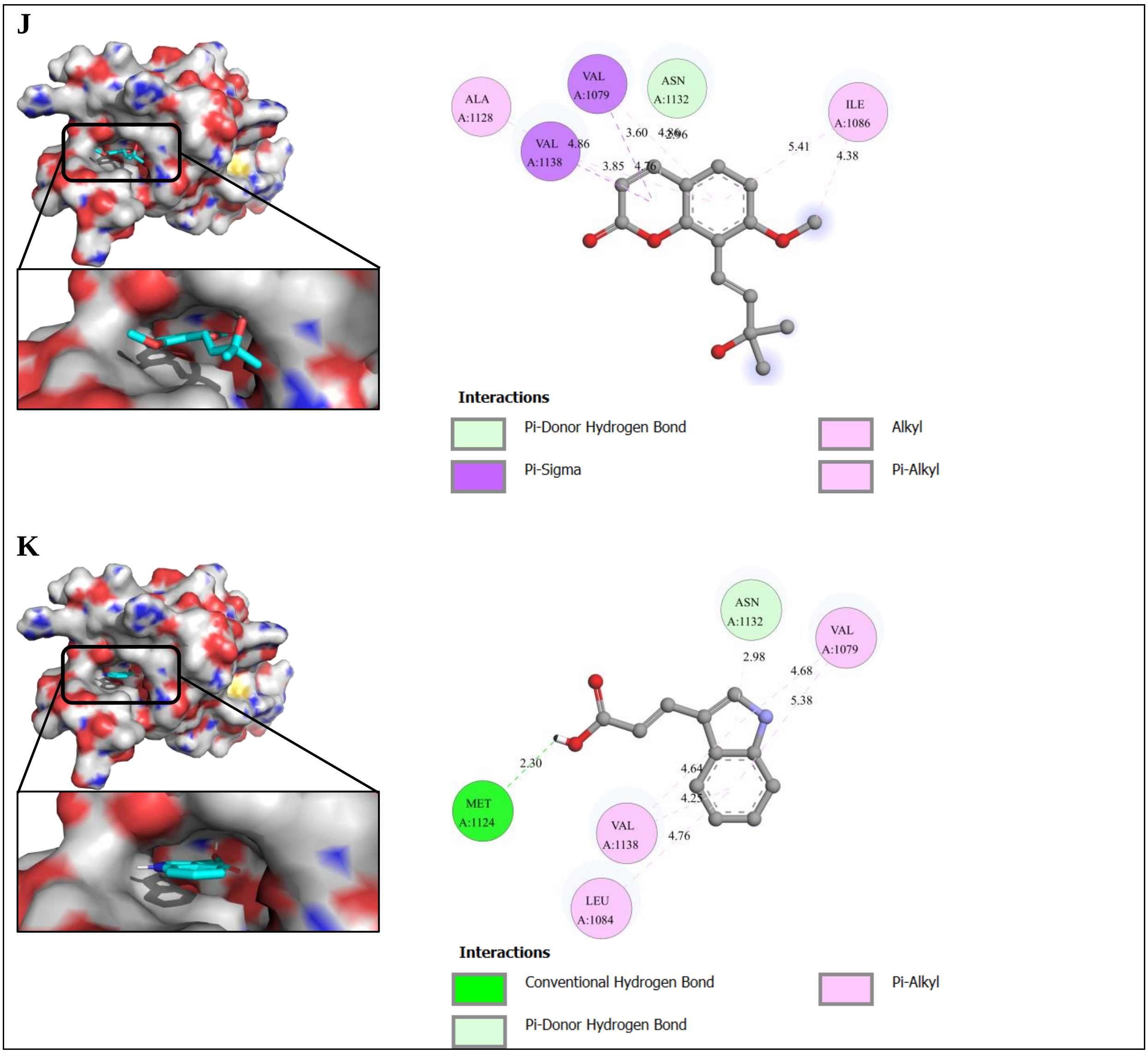
J
VAL A:1079
ASN A:1132
ALA A:1128
ILE A:1086
VAL A:1138
4.86
3.60
3.85
4.76
5.41
4.38
Interactions
Pi-Donor Hydrogen Bond
Alkyl
Pi-Sigma
Pi-Alkyl
K
ASN A:1132
VAL A:1079
2.98
4.68
5.38
2.30
MET A:1124
4.64
VAL A:1138
4.76
LEU A:1084
Interactions
Conventional Hydrogen Bond
Pi-Alkyl
Pi-Donor Hydrogen Bond
