# Supplementary figures and images for: Unveiling the anti-obesity potential of Kemuning (Murraya paniculata): A network pharmacology approach
Source: PLoS One. 2024 Aug 29;19(8):e0305544. doi: 10.1371/journal.pone.0305544 (PMC11361609; doi:10.1371/journal.pone.0305544)

### S3 File. The RMSD graphs of all ligands compared to the native ligands of PPARG

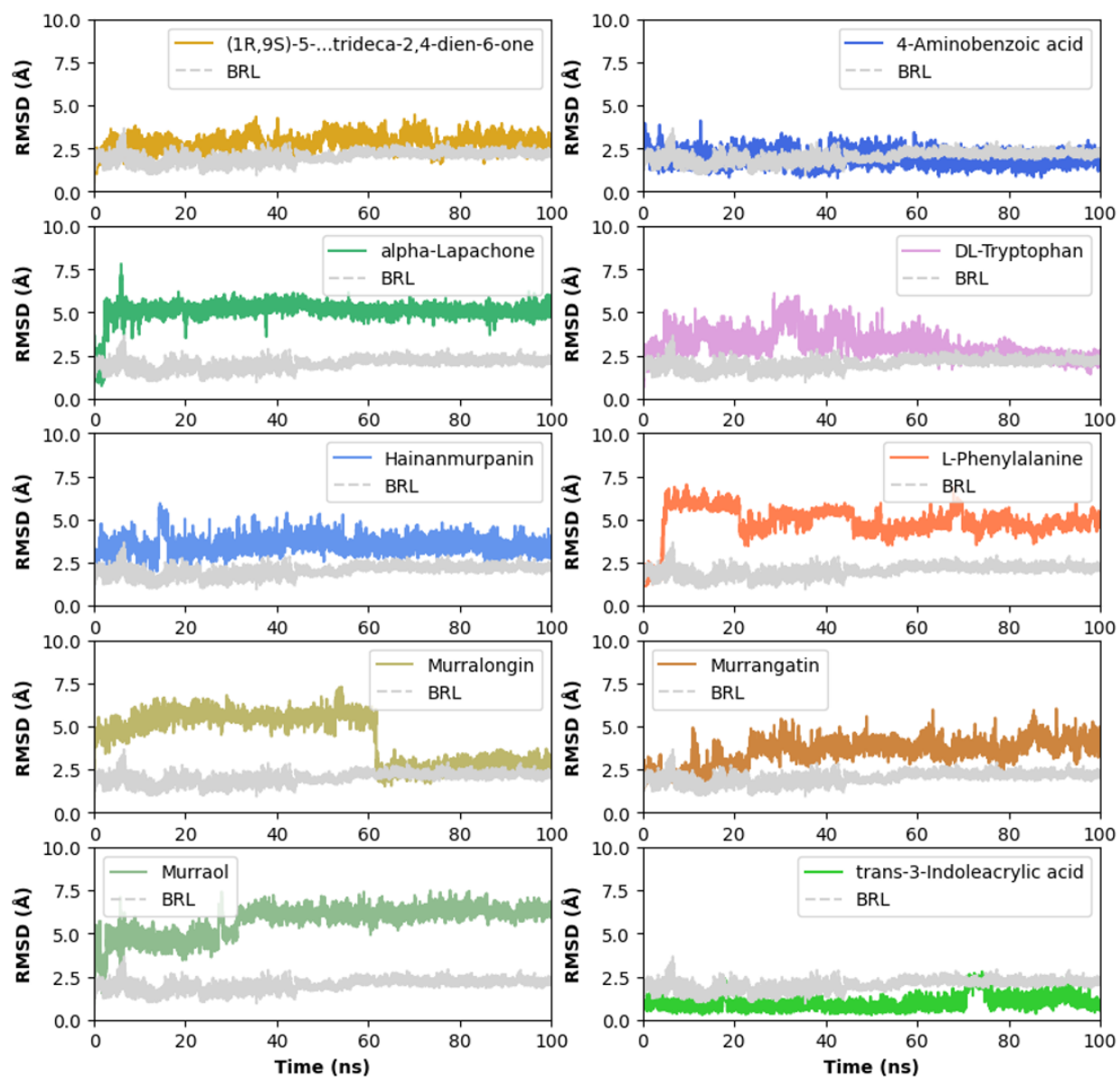

Supplement: S3 File — (PDF) [file pone.0305544.s012.pdf]

**S4 File. The RMSD graphs of all ligands compared to the native ligands of EP300**

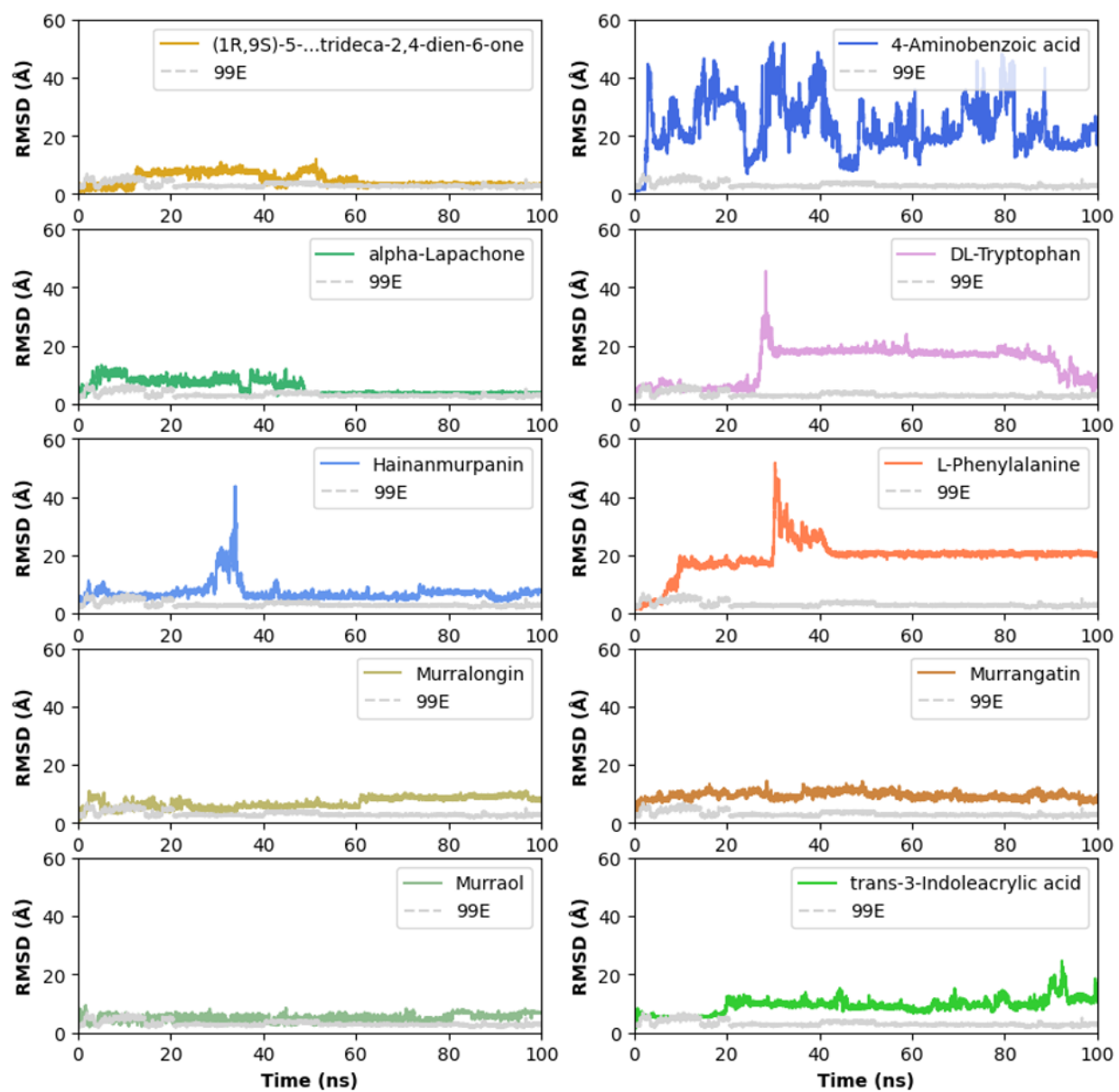

Supplement: S4 File — (PDF) [file pone.0305544.s013.pdf]

**S5 File. The RMSF graphs of PPARG in all ligands and native ligands-bound structures**

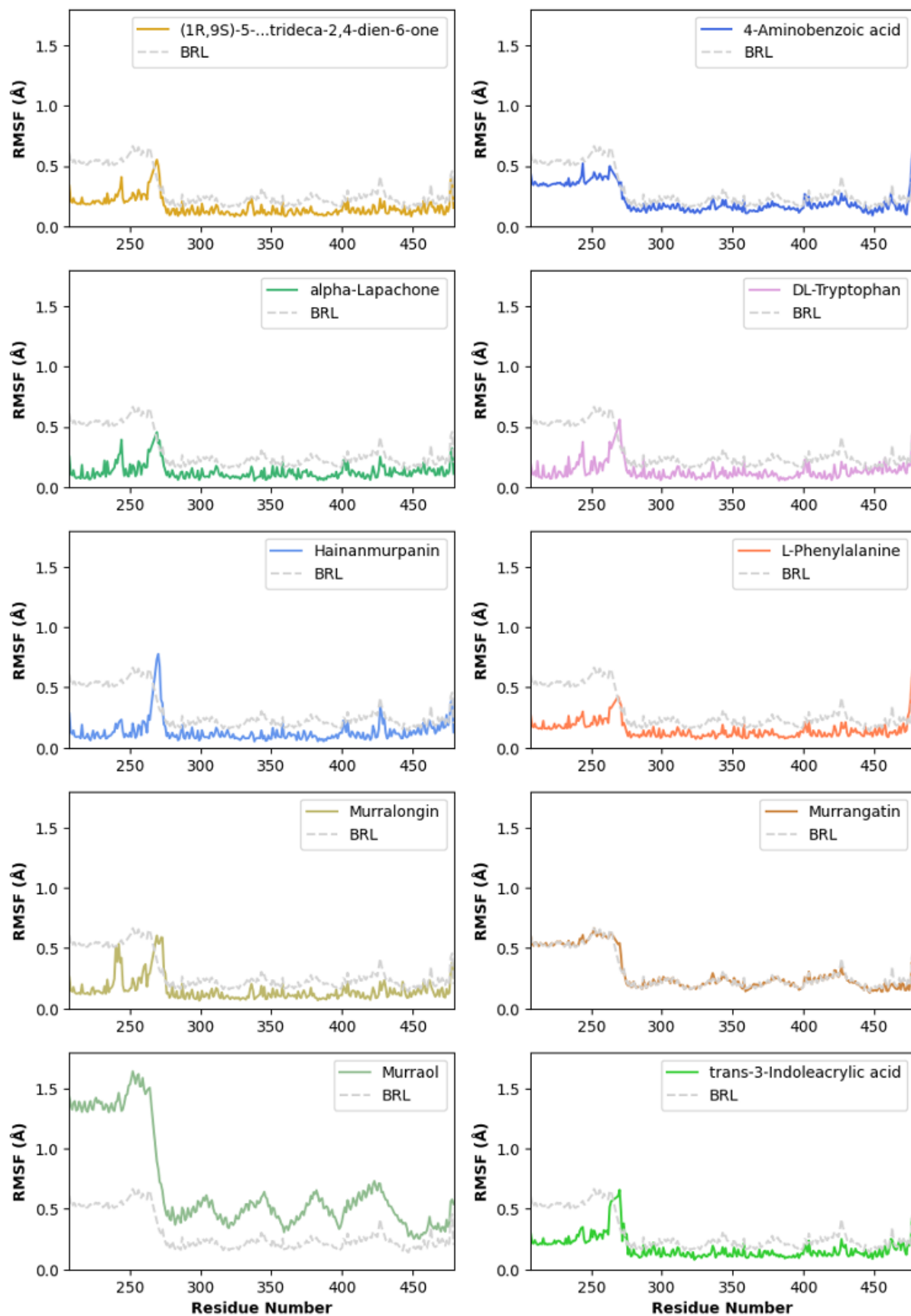

Supplement: S5 File — (PDF) [file pone.0305544.s014.pdf]

**S6 File. The RMSF graphs of EP300 in all ligands and native ligands-bound structures**

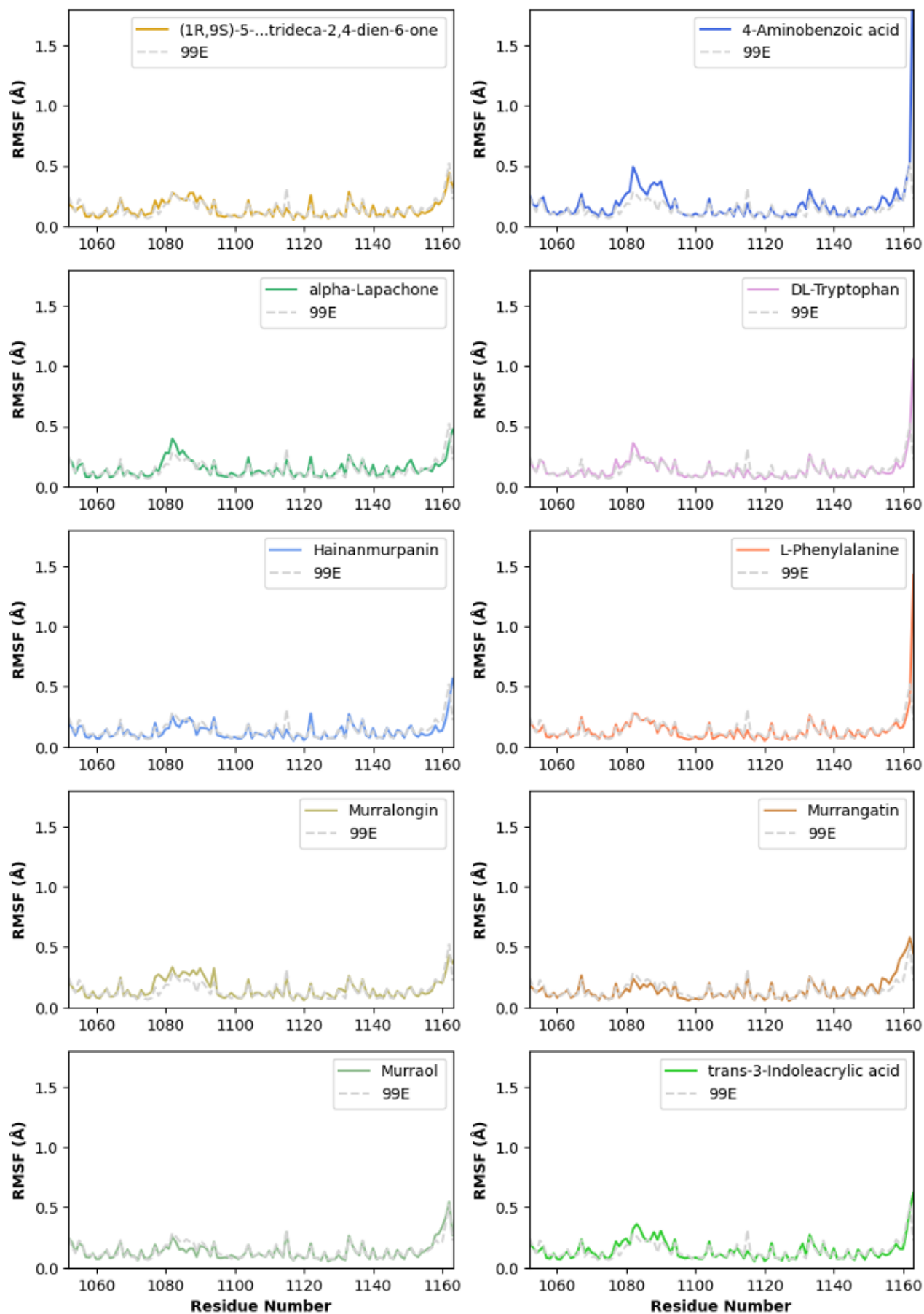

Supplement: S6 File — (PDF) [file pone.0305544.s015.pdf]
